# Necroptosis-associated long noncoding RNAs can predict prognosis and differentiate between cold and hot tumors in ovarian cancer

**DOI:** 10.3389/fonc.2022.967207

**Published:** 2022-07-28

**Authors:** Yi-bo He, Lu-wei Fang, Dan Hu, Shi-liang Chen, Si-yu Shen, Kai-li Chen, Jie Mu, Jun-yu Li, Hongpan Zhang, Liu Yong-lin, Li Zhang

**Affiliations:** ^1^ Department of Clinical Lab, The First Affiliated Hospital of Zhejiang Chinese Medical University, Hangzhou, China; ^2^ The First School of Clinical Medicine, Zhejiang Chinese Medical University, Hangzhou, China; ^3^ Department of Clinical Lab, The Cixi Integrated Traditional Chinese and Western Medicine Medical and Health Group Cixi Red Cross Hospital, Cixi, China; ^4^ Department of Pharmacy, Sanya Women and Children Hospital Managed by Shanghai Children’s Medical Center, Sanya, China; ^5^ Department of Oncology, Affiliated Hospital of North Sichuan Medical College, Nanchong, China; ^6^ Reproductive Centre, Sanya Women and Children Hospital Managed by Shanghai Children’s Medical Center, Sanya, China; ^7^ Obstetrics and Gynaecology, The First Affiliated Hospital of Zhejiang Chinese Medical, Hangzhou, China

**Keywords:** ovarian cancer, necroptosis, immunotherapy, long noncoding RNAs, TCGA

## Abstract

**Objective:**

The mortality rate of ovarian cancer (OC) is the highest among all gynecologic cancers. To predict the prognosis and the efficacy of immunotherapy, we identified new biomarkers.

**Methods:**

The Cancer Genome Atlas (TCGA) and the Genotype-Tissue Expression Project (GTEx) databases were used to extract ovarian cancer transcriptomes. By performing the co-expression analysis, we identified necroptosis-associated long noncoding RNAs (lncRNAs). We used the least absolute shrinkage and selection operator (LASSO) to build the risk model. The qRT-PCR assay was conducted to confirm the differential expression of lncRNAs in the ovarian cancer cell line SK-OV-3. Gene Set Enrichment Analysis, Kaplan-Meier analysis, and the nomogram were used to determine the lncRNAs model. Additionally, the risk model was estimated to evaluate the efficacy of immunotherapy and chemotherapy. We classified necroptosis-associated IncRNAs into two clusters to distinguish between cold and hot tumors.

**Results:**

The model was constructed using six necroptosis-associated lncRNAs. The calibration plots from the model showed good consistency with the prognostic predictions. The overall survival of one, three, and five-year areas under the ROC curve (AUC) was 0.691, 0.678, and 0.691, respectively. There were significant differences in the IC50 between the risk groups, which could serve as a guide to systemic treatment. The results of the qRT-PCR assay showed that AL928654.1, AL133371.2, AC007991.4, and LINC00996 were significantly higher in the SK-OV-3 cell line than in the Iose-80 cell line (P < 0.05). The clusters could be applied to differentiate between cold and hot tumors more accurately and assist in accurate mediation. Cluster 2 was more vulnerable to immunotherapies and was identified as the hot tumor.

**Conclusion:**

Necroptosis-associated lncRNAs are reliable predictors of prognosis and can provide a treatment strategy by screening for hot tumors.

## Introduction

The mortality rate of ovarian cancer (OC) is the highest among all gynecologic cancers. In developed countries, nearly 145,000 new cases and 100,000 deaths are reported each year (1). Since the symptoms usually appear late, OC is often detected at an advanced stage (2). For advanced ovarian cancer, median progression-free survival (PFS) and overall survival (OS) have a range of 12 – 24 months and 29 – 65 months, respectively (3). The proportion of deaths due to ovarian cancer has remained constant over time. Since the prognosis for OC patients is poor and the efficacy of conventional approaches is limited, new therapeutic strategies are essential. The advent of immunotherapy has significantly changed treatment across many malignancies (4). However, most cancer patients fail to respond to immunotherapies such as blockade of PD-1, PD-L1, and CTLA-4 immune checkpoint axis antibodies (5). Therefore, ways to enhance the effectiveness of immunotherapy in ovarian cancer need to be investigated.

Due to the resistance of tumors to apoptosis, many other cell death mechanisms, including necroptosis, are considered to be advanced therapeutic strategies (6). Necroptosis is an alternative program of necrotic cell death to apoptosis and activates RIPK1 and RIPK3 within the tumor microenvironment. This causes an increase in CD8^+^ leukocyte-mediated antitumor immunity (7). Moreover, necroptosis promotes malignancies by activating the immune suppressants Mincle and CXCL1, suggesting that necroptosis is a latent target for immunotherapy in OC (8).

LncRNA (long noncoding RNA) consists of more than 200 nucleotides but cannot code for proteins (9). The necroptosis of hepatocellular carcinoma cells is caused by the release of miRNAs related to Linc00176, e.g., miR-9 and miR-185 (10). TRINGS is a P53-inducible lncRNA that protects tumor cells from necrotic apoptosis by inhibiting TRAPGSK3β-NF-κB necroptosis signaling (11). In cardiomyocytes, miR-873 and RIPK1/RIPK3 are targeted by the lncRNA necrosis-related factor (NRF) (12). Necroptosis-associated lncRNAs have not been investigated as potential therapeutic targets in ovarian cancer. Thus, studying necroptosis-associated lncRNAs might elucidate how necroptosis and lncRNAs influence immunotherapy of OC.

Tumors infiltrated by immune cells can be either “hot” or “cold”, depending on the degree of infiltration. In contrast to immunological “hot” tumors that have high T cell infiltration, “cold” tumors have little or no T cells or are confined to the periphery of the tumor (13). The discovery of “hot tumors” might result in a breakthrough in immunotherapy, while other mechanisms of cell death in OC remain partially determined. However, a simple and effective way to distinguish between tumors remains unknown (14). Considering that lncRNAs are promising cancer biomarkers, we grouped patients by necroptosis-associated lncRNAs. Early identification of hot tumors can improve the prognosis and enhance precision mediation in clinical practice (15).

## Materials and methods

### Data processing

The RNA-Seq and the corresponding clinical data of OC patients were obtained from GTEx (https://www.gtexportal.org/) (version 10, February 2022) and TCGA (https://portal.gdc.cancer.gov/) (version 10, February 2022). Two synthetic data matrices were obtained. To determine the differential expression of lncRNAs, we used the count value matrix, and for other analyses, we used the FPKM value matrix. To decrease statistical bias, we excluded OC patients with missing or small values of overall survival (OS) (<30 days). We combined the relevant clinical data to retrieve data on 365 patients and randomized them into training and testing risk groups in a 1:1 ratio using the R package “caret”.

### Necroptosis-associated genes and screening for lncRNAs

The gene set M24779.gmt comprises eight necroptosis genes and is available for download from Gene Set Enrichment Analysis (GSEA) (http://www.gsea-msigdb.org/gsea/index.jsp). Furthermore, we obtained a profile of 67 necroptosis-associated genes combining previous reports on necroptosis (**Appendix T1**). We performed a correlation analysis of necroptosis-associated genes and the differential expression of lncRNAs in the matrices. We found that 54 necroptosis-associated genes were associated with 385 lncRNAs (Pearson’s correlation coefficient >0.4, p < 0.001). We considered these 385 IncRNAs to be necroptosis lncRNAs.

### Risk prediction signature model

Based on clinical data on OC patients from the GTEx and TCGA, survival-associated lncRNAs from necroptosis lncRNAs were obtained by performing a univariate Cox proportional risk regression analysis (P < 0.05). Cross-validated Lasso regressions were performed for 1,000 cycles with a p-value of 0.05. A random simulation was run 1,000 times to avoid overfitting for each cycle. Then, a risk prediction model was constructed. Receiver operating characteristic (ROC) curves for one, three, and five years with time were plotted using the “time ROC” R package. The risk score was calculated using the following formula (16):

$risk\;score\;=\sum_{k=1}^{n}coef\left(IncRNA^{k}\right)*expr\left(IncRNA^{k}\right)$ (1), where coef(lncRNA) denoted the coefficient of lncRNAs associated with survival and expr(LncRNAn) was the expression of all lncRNAs. A median risk score was used to divide the results into low-risk and high-risk groups (17, 18). A Chi-squared test was performed to obtain a prognostic value for the risk signature model by examining its correlation with the clinical characteristics.

### Independence factors and ROC

A univariate Cox (uni-Cox) regression and multivariate Cox (multi-Cox) regression analysis identified the independent risk factors. The ROC was determined to compare different factors involved in predicting outcomes.

### Nomogram and calibration

The Hosmer-Lemeshow test was used to construct nomograms of one-, three-, and five-year OS and calibration curves according to the risk score, age, and tumor stage. Nomograms were constructed to determine whether the predicted result and the actual outcome were consistent. This analysis was performed using the R package “RMS”.

### KEGG enrichment analyses

Gene set enrichment analyses (GSEA) were conducted using the GSEA software (http://software.broadinstitute.org/gsea/index.jsp) to find enriched KEGG pathways between the low-risk and high-risk groups according to the criteria of p < 0.05 and FDR < 0.25.

### Correlation between immune markers and immune checkpoints

We examined the immune cell factors in the high-risk group. The immune infiltration status of the GC patients in the TCGA database was assessed using the TIMER2.0 online tool (http://timer.cistrome.org/). Additionally, we downloaded the infiltration estimation profiles for all TCGA tumors from the same site. Using the Wilcoxon signed-rank test and the R packages “ggplot2”, “Scale,” “ggText”, and “LIMMA”, the differences in the content of immune infiltrating cells were analyzed. Bubble plots were used to visualize the results (17). In this study, we compared the TME scores and immune checkpoint activation of the low-risk and high-risk groups using the R package “ggpubr”.

### Drug sensitivity analysis

The half-maximal inhibitory concentration (IC50) for each OC patient was calculated from Genomics of Drug Sensitivity in Cancer (https://www.cancerrxgene.org/), and the R package “pRRophetic” was used to predict the therapeutic response.

### Validation of lncRNA in the risk model by qRT-PCR

The human normal ovarian epithelial cell line Iose-80 and the ovarian cancer cell line SK-OV-3 were obtained from the Cell Bank of the Chinese Academy of Medical Sciences. Total RNA from the cell lines was isolated using the RNeasy mini kit (Servicebio, China). The primers used for PCR amplification are presented in **Table 1**. Three replicates of each sample were performed, and GAPDH was used as a control. The relative expression levels were determined using the 2^-ΔΔCT^ method. The differences in the expression of AP003392.3, AL928654.1, AL133371.2, AC007991.4, AC011445.1, and LINC00996 were determined by conducting t-tests. The graphs were constructed using GraphPad Prism (version 7.0.2) (* indicates p < 0.05)

**Table 1:** Primer sequences in our study.

Primer	Forward (5’-3’)	Reverse (5’-3’)	Fragments (bp)
**AP003392.3**	AGGGACTCACAGTAGAAAGCACA	AATGGAAACTGTTCTCCTCCTCT	114
**AL928654.1**	TGTGGAAAATTCAGTGGGAACA	GCTGGTAGAAACAGGAGGGAGT	123
**AL133371.2**	ATTGGGAAGAGATTAGCAGGTCAG	AGATTCTCCCTGCCATTCCAC	85
**AC007991.4**	CAGAACCAAAGCCAGTAAATCCT	CGACTGTTTGGAGAGTTACATTACC	194
**AC011445.1**	TTCTCAGCCTTGCCGCTT	ACAACTCCCGTTTATTGACAGC	123
**LINC00996**	GAGCTTAGACCTGCTTCCACTTTC	TGCTTCATCAGGCTGTTGTGG	142
**GAPDH**	GGAAGCTTGTCATCAATGGAAATC	TGATGACCCTTTTGGCTCCC	168

### Clusters based on prognostic lncRNAs

The R package “ConensusClusterPlus(CC)” was used to discover potential molecular subgroups according to the prognostic expression of lncRNAs (19). Kaplan–Meier survival, T-distributed stochastic neighbor embedding (T-SNE), and Principal component analysis (PCA) were performed using the R package Rtsne. The GSEA was performed to identify enriched immunologic signatures between clusters using the criteria of p < 0.05 and FDR < 0.25 (20). Immunoassays and drug sensitivity of different clusters were performed using the R packages “GSVA Base” and “pRRophetic “.

## Results

### Necroptosis-associated lncRNAs in OC patients

**Figure 1:**
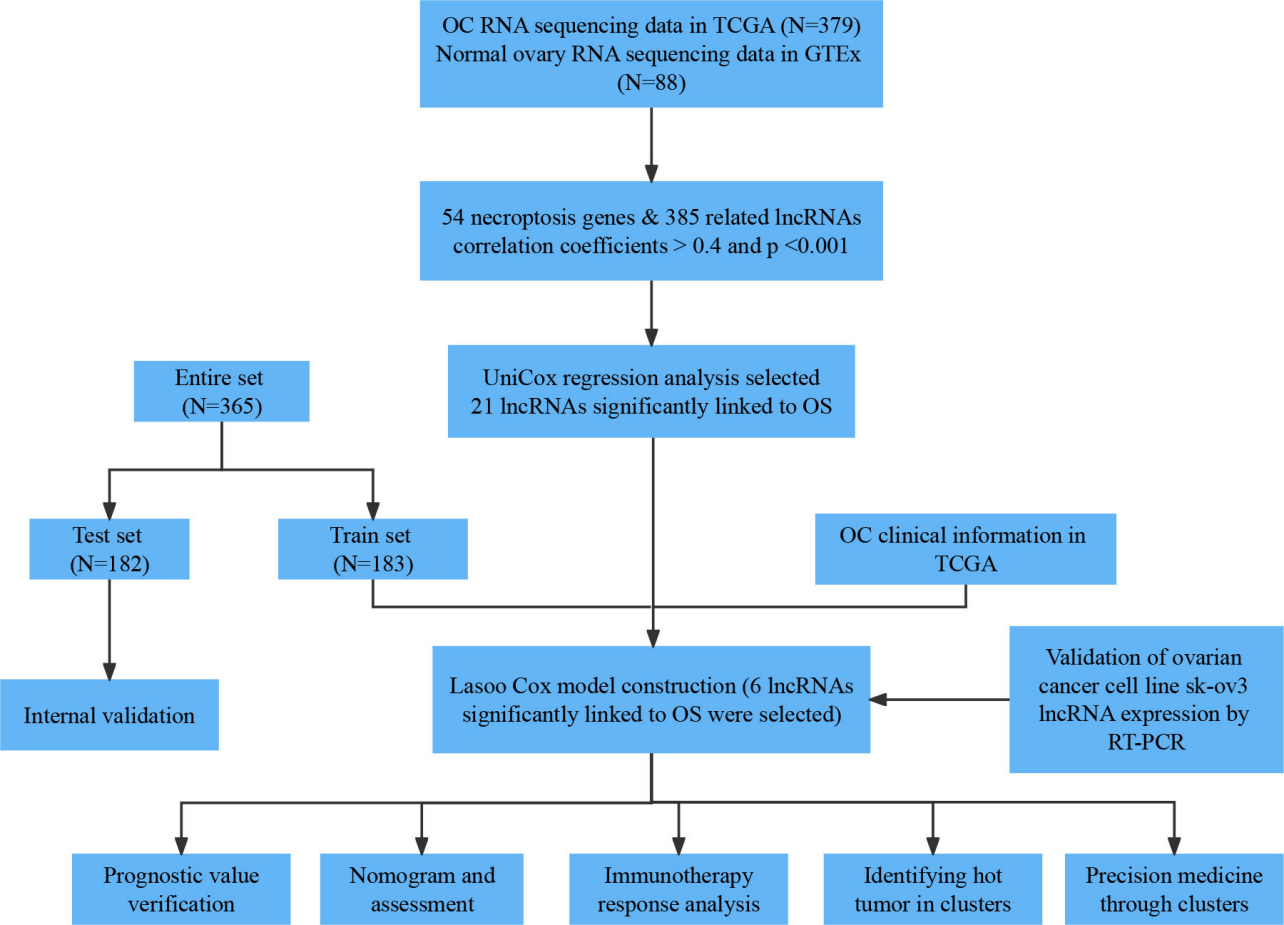
Flow chart of our research.

**Figure 2:**
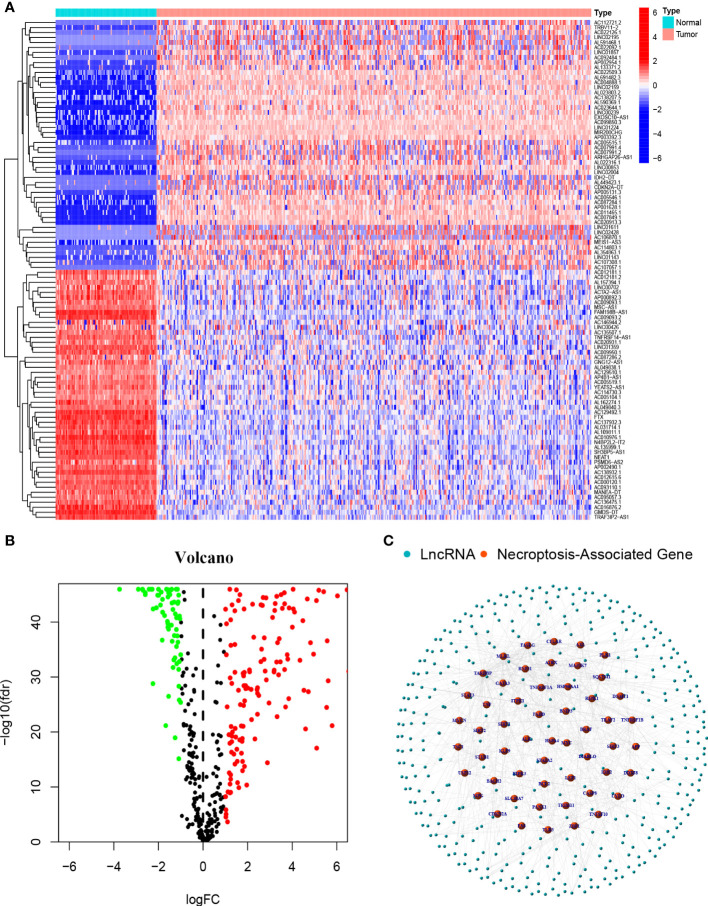
Necroptosis-associated Genes and lncRNAs screening in patients with OC **(A)** The heatmap of differentially expressed necroptosis-associated lncRNAs. **(B)** Volcano plot of 387 differentially expressed necroptosis-associated lncRNAs. **(C)** The network between necroptosis genes and lncRNAs (correlation coefficients>0.4 and p<0.001).

A flowchart of our study is shown in **Figure 1**. We obtained 373 tumor samples from TCGA and 112 normal samples from GTEx. Based on the expression analysis of 67 necroptosis genes and differentially expressed lncRNAs (|Log_2_FC| > 1 and p < 0.05), we found that 54 of these necroptosis genes were associated with 385 IncRNAs (correlation coefficient > 0.4 and p < 0.001). These 385 IncRNAs were identified as necroptosis IncRNAs (16, 17). Among these IncRNAs, 145 were upregulated, while the rest were downregulated (**Figures 2A, B**). The network plot showed the relationship between the necroptosis genes and the IncRNAs (**Figure 2C**). More details can be found in **Appendix D1**.

### Risk model construction and verification

**Figure 3:**
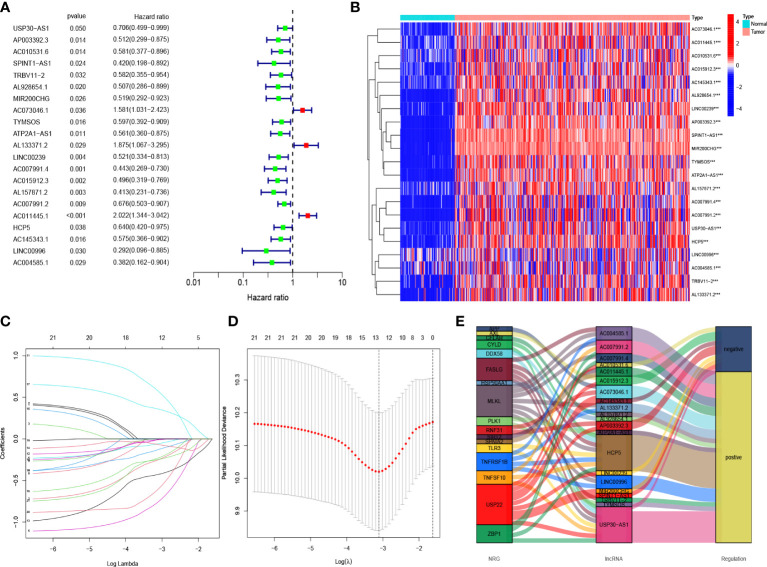
Risk prediction signature model in patients with OC **(A)** The prognostic lncRNAs obtained with uni-Cox regression analysis. **(B)** The heatmap of differentially expressed lncRNAs. **(C)** In the LASSO model, the 10-fold cross-validation for variable selection. **(D)** Cross-validation of error curves is performed with the tuning parameters (log λ) of patients’ OS-related lncRNAs. The imaginary perpendicular line is also dragged to the excellent value. **(E)** Necroptosis genes and lncRNAs are shown in the Sankey diagram.

The results of the univariate COX regression analysis showed that 21 necroptosis-associated lncRNAs were significantly associated with OS (p < 0.05), and a heat map was constructed to show the gene expression density (**Figures 3A**, **B**). Lasso regression was performed on these lncRNAs and 21 necroptosis lncRNAs were extracted to avoid overfitting the prognostic signature. The first rank value of log (λ) was the least probable deviation (**Figures 3C**, **D**). Based on the Sankey plots, 14 upregulated lncRNAs and seven downregulated lncRNAs were identified (**Figure 3E**).

**Figure 4:**
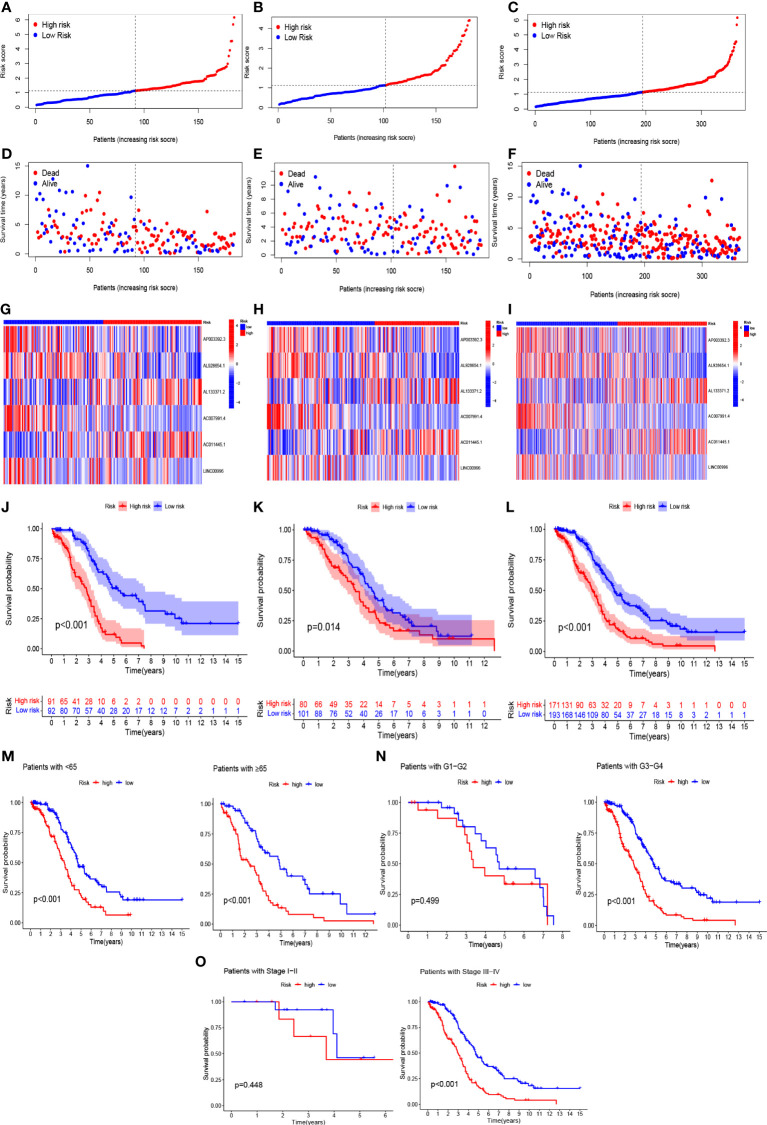
In the train, test, and entire sets, the prognostic value of the model for six necroptosis-associated lncRNAs. **(A–C)** A model of necroptosis-associated lncRNAs according to risk score of the train, test, and entire sets is displayed, respectively. **(D–F)** Surviving time and survival status among low- and high-risk groups in the train, test, and overall sets. **(G–I)** The heat maps of 6 lncRNAs expression can be seen in the train, test, and overall set. **(J–L)** Overall survival of OC patients in the train, test, and entire sets between low- and high-risk groups, respectively **(M–O)** Survival curves of Kaplan–Meier of OS prognostic value based on age, grade, and stage between low- and high-risk groups in the entire set.

Based on the results of lasso regression, six lncRNAs were used to establish the risk model. The formula for calculating the risk score was as follows: *Risk score* = *AP*003392.3 × (-0.4792)+*AL*928654.1 × (-1.2002) + *AL*133371.2 × (0.9982) + *AC*007991.4 × (-0.7437) + *AC*011445.1 × (0.5334) + *LIN*C00996 × (-1.454) (2) (16, 17). The OC samples were considered to be low-risk and high-risk groups according to the median value of the prognostic risk grade. We compared the survival status and survival time of the low-risk and high-risk groups in the training, testing, and entire sets (**Figures 4A**–**I**). The results indicated that OS was significantly lower in the high-risk group than in the low-risk group (p < 0.05) (**Figures 4J**–**L**). OS was lower in the high-risk group than in the low-risk group for patients of different ages. In the tumor grade and stage subgroup, neither G1-G2 nor stage I-II differed significantly in overall survival (p > 0.05). However, in G3-G4 and stage III-IV, OS was significantly lower in the high-risk group than in the low-risk group (p < 0.001) (**Figures 4M**–**O**).

### Formation and assessment of the nomogram

**Figure 5:**
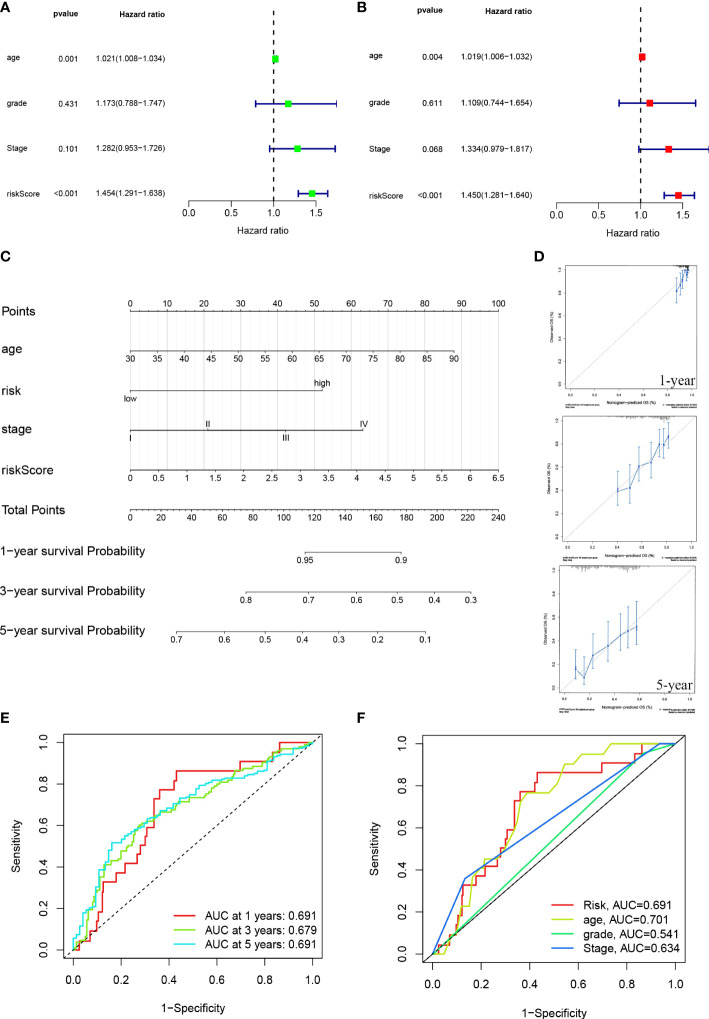
ROC diagram and nomogram for the risk model. **(A, B)** Uni-Cox and multi-Cox analyses of risk score and clinical Characteristics with OS. **(C)** The probability of the 1-, 3-, and 5-year OS was predicted by combining the nomogram with the risk, risk score, age, and stage. **(D)** The calibration curves for 1-, 3-, and 5-year OS. **(E)** The risk model’s 1-, 3-, and 5-year ROC curves. **(F)** Five-year ROC curves of risk score and clinical characteristics.

This hazard ratio (HR) and 95% confidence interval (CI) for risk scores were 1.454 and 1.291 – 1.638 (p < 0.001) in the univariate COX regression and 1.450 and 1.281 – 1.640 (p < 0.001) in the multivariate COX regression, respectively (**Figures 5A**, **B**). We constructed a nomogram, including risk scores and clinical characteristics, to predict overall survival at one, three, and five years. The risk scores of the risk model revealed that the nomogram could accurately predict by comparing the clinical characteristics (**Figure 5C**). The correction chart suggested that the measured and predicted values for the one-, three-, and five-year OS indicated an ideal consistency (**Figure 5D**).

### Risk model assessment

The sensitivity and specificity of the prognostic prediction model were assessed using time-dependent receiver operating characteristics (ROC). The area under the ROC curve (AUC) was used to present the results of the ROC. The one-, three-, and five-year AUC were 0.691, 0.679, and 0.691 (**Figure 5E**). Regarding the five-year ROC of the risk model, the AUCs of risk, age, grade, and stage were 0.691, 0.701, 0.541, and 0.639, respectively (**Figure 5F**).

### GSEA

**Figure 6:**
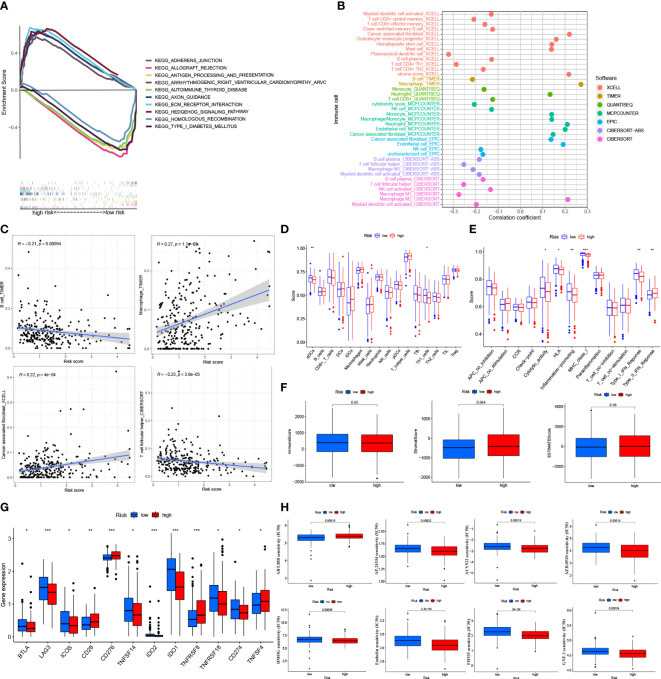
Tumor immune factors and drug sensitivity analysis in risk model **(A)** Kegg Pathway analysis between low- and high-risk. **(B)** The bubble plot of immune cells in the risk model. **(C)** The Relationship between immune cells and risk score **(D)**. **(E)** The ssGSEA scores for immune cells and immune function in the risk groups. **(F)** The association of immune-related scores between low- and high-risk groups. **(G)** Immune checkpoints expression in risk groups. **(H)** Drug sensitive analysis in the risk model. *: p < 0.05. **: p < 0.01. ***: p < 0.0001.

We examined the low-risk and high-risk groups in the KEGG pathway across the entire set using the GSEA software to determine the differences in biological function between the different risk groups (**Supplementary Figure 1**). Gene Set Enrichment Analysis, KEGG_AXON_GUIDANCE, KEGG_ ADHERENS_JUNCTION, KEGG_HEDGEHOG_SIGNALING_PATHWAY, KEGG_ ECM_ RECEPTOR_INTERACTION, and KEGG_ARRHYTHMOGENIC_RIGHT_VENTRICULAR_ CARDIOMYOPATHY_ARVC were significantly correlated with the high-risk group (p < 0.001). KEGG_ ANTIGEN_PROCESSING_AND_PRESENTATION, KEGG_AUTOIMMUNE_ THYROID_DISEASE, KEGG_TYPE_I_DIABETES_MELLITUS, KEGG_ALLOGRAFT_ REJECTION, and KEGG_HOMOLOGOUS_RECOMBINATION were significantly correlated with the low-risk group (p < 0.001) (**Figure 6A**).

### Immune signature in risk groups

On different platforms, there were many immune cells associated with the risk groups (p < 0.05) (**Figure 6B** and **Appendix D2**). Immune cells like T follicular helper cells, B cells, macrophages, and cancer-associated fibroblasts were associated with risk scores that significantly affected the immunotherapy of tumors (p < 0.001) (**Figure 6C** and **Supplementary Figure 2A**) (21). By the ssGSEA method, most immune cells did not differ significantly between the risk groups (p > 0.05) (**Figure 6D**). Some immune cell pathways showed higher scores in the low-risk group (p > 0.05) (**Figure 6E**). Based on the TME (tumor microenvironment) evaluation system, the high-risk group had a higher Stromal Score than the low-risk group (p < 0.05). However, the low-risk and high-risk groups did not differ significantly from each other regarding the Immune Score and the Estimates Score (p > 0.05) (**Figure 6F**). In the immune checkpoints analysis, the expression of the BTLA, LAG3, ICOS, TNFSF14, IDO2, IDO1, and TNFSF18 genes was higher in the low-risk group, while the expression of the CD28, CD276, TNFSF8, and TNFSF14 genes was higher in the high-risk group (p < 0.05) (**Figure 6G**). The IC50 of 19 drugs was significantly different between the low-risk and high-risk groups. For most medicines, the IC50 values were higher in the low-risk group of patients (p < 0.05; **Figure 6H** and **Supplementary Figure 2B**).

### Validation of lncRNA in the risk model

**Figure 7:**
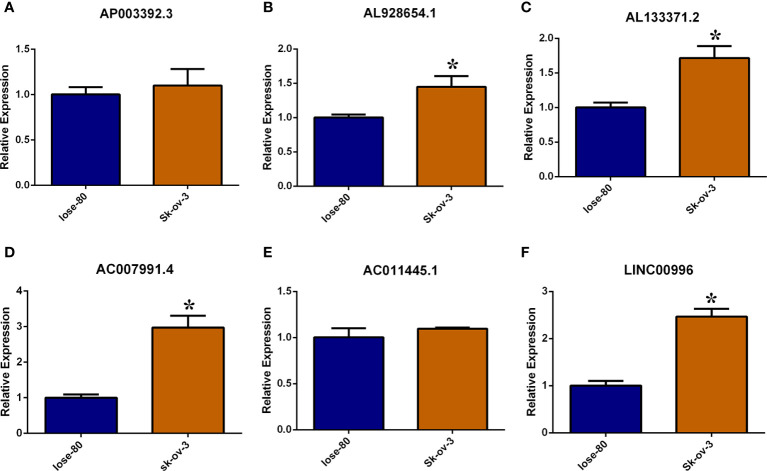
Validate necroptosis-associated lncRNAs in risk model by RT-PCR method. **(A–F)** Relative expression of AP003392.3, AL928654.1, AL133371.2, AC007991.4 AC011445.1 and LINC00996 in Iose-80 and Sk-ov-3 cell line. *: p < 0.05.

The necroptosis-associated lncRNAs were selected in the risk model (AP003392.3, AL928654.1, AL133371.2, AC007991.4 AC011445.1, and LINC00996). These lncRNAs were tested in the Iose-80 and SK-OV-3 cell lines. The results indicated that the expression of AL928654.1, AL133371.2, AC007991.4, and LINC00996 was significantly higher in the SK-OV-3 cell line than in the Iose-80 cell line (p < 0.05) (**Figures 7B**–**D**, **F**), which was consistent with the results from the GETx and TCGA databases. However, the expressions of AP003392.3 and AC011445.1 did not differ significantly between the two cell lines (p > 0.05) (**Figures 7A**, **E**).

### Cold and hot tumor cluster screening

**Figure 8:**
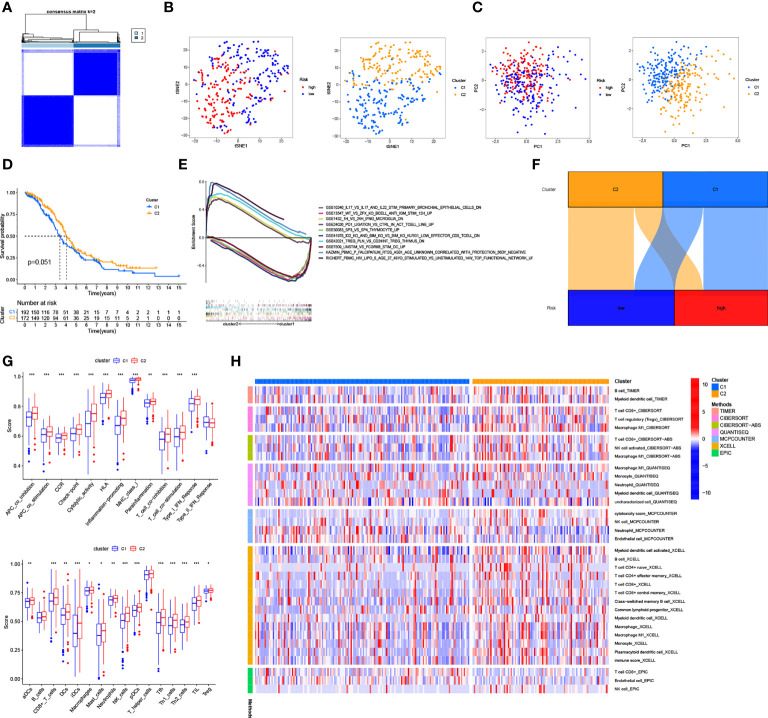
Cold and Hot Tumor Cluster Screening. **(A)** According to ConsensusClusterPlus, OC patients are split into two clusters. **(B)** Risk groups and clusters of T-SNE. **(C)** The PCA for risk groups and clusters. **(D)** Survival curves of Kaplan–Meier for OS in clusters. **(E)** The GSEA of immunologic signature in clusters. **(F)** The Sankey diagram of risk groups and clusters. **(G)** The ssGSEA scores in clusters. **(H)** The heat map shows immune cells grouped in clusters. *: p < 0.05. **: p < 0.01. ***: p < 0.0001.

**Figure 9:**
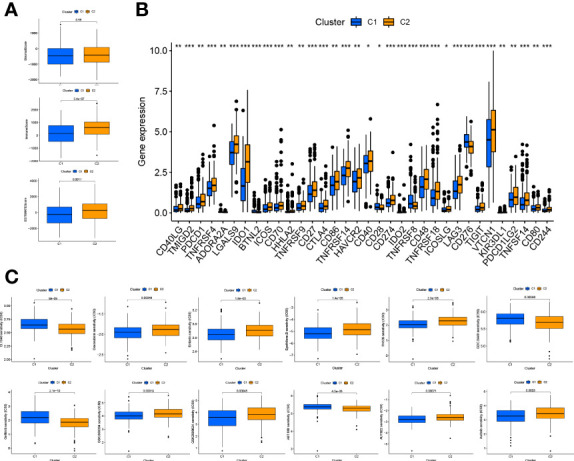
Immunoassay and drug sensitivity analysis in clusters **(A)** The relationship between immune-related scores in cluster1 and cluster2. **(B)** Immune checkpoints expression in risk groups. **(C)** Drug sensitive analysis in clusters. *: p < 0.05. **: p < 0.01. ***: p < 0.0001.

Based on the expression of the six necroptosis-associated lncRNAs and OS of OC patients, the non-negative matrix factorization was performed to categorize the OC samples into two clusters (cluster 1 and cluster 2) using the R package “ConsensusClusterPlus” (CC) (**Figure 8A** and **Supplementary Figure 3A**). Different clusters exhibit different immune microenvironments, resulting in different reactions to immunotherapeutic responses (22, 23). The two clusters could be distinguished according to T-distributed stochastic neighbor embedding (T-SNE) and were significantly more differentiated than the high-risk and low-risk groups. (**Figure 8B**) Moreover, principal component analysis (PCA) was performed to verify that the risk and cluster groups had different principal components (**Figure 8C**). Based on the results of the Kaplan–Meier analysis, cluster 2 presented a better overall survival than cluster 1 (p = 0.051) (**Figure 8D**). GSEA was used to investigate the immunological function of the clusters (**Figure 8E** and **Supplementary Figure 3A**). Cluster 1 was related to the low-risk group, while cluster 2 was related to the high-risk group based on the Sankey diagram (**Figure 8F**). The ssGSEA score indicated that the immune cells, including CD8^+^ T cells, and the immune functions, including pro-inflammatory function, were more associated with cluster 2 (**Figure 8G**). The results of the analysis of the different platforms showed that cluster 2 had a higher degree of immune infiltration (**Figure 8H**) (**Appendix D3**). The immune score and estimated (microenvironment) score were higher in cluster 2, suggesting a different TME from cluster 1 (**Figure 9A**). Nearly all immune checkpoints, including LAG3, CD274 (PD-L1), and HAVCR2 (TIM3), were significantly higher in cluster 2 than in cluster 1 (**Figure 9B**). Hot tumors had more CD8^+^ T cells, pro-inflammatory functions, and activation of immune checkpoints like TIM3, LAG3, and PD-L1 (14). Therefore, we classified cluster 2 as hot tumors and cluster 1 as cold tumors. Hot tumors were sensitive to immunotherapy, while cold tumors were resistant to immunotherapy (14, 23). Based on the concept of cold and hot tumors, cluster 2 was more sensitive to immunotherapy. We also found significant differences in the IC50 of 36 drugs between the clusters, with most drugs having a higher IC50 in cluster 2 (p < 0.05) (**Figures 9C** and **Supplementary Figure 3C**). Based on the cluster of necroptosis-associated lncRNAs, we might further investigate the immunotherapeutic and therapeutic drug response in OC patients.

## Discussion

The commonly reported treatment failures might be reduced by immunotherapy, but it is not a panacea for all illnesses (23). Several patients did not respond well to immunotherapy because of immunosuppressive TME (24). To improve the effectiveness of immunotherapy, we incorporated the concept of cold and hot tumors based on the immune response rather than the conventional cancer classification of tumors. Typically, highly infiltrative tumors with a high immune score are called hot tumors, while non-infiltrative tumors with a low immune score are called cold tumors (14). Additionally, hot tumors also exhibit higher checkpoint activities, such as TIM3 and LAG3. Patients with hot tumors can be treated with immunotherapies that target T cells, microbiome modulations, or other immunotherapies. The treatment of patients with “cold” tumors (those un-infiltrated with CD8^+^ T cells) is ineffective, indicating the need to develop new ways to convert cold tumors into receptive hot tumors (14). Numerous pathogenic processes include necroptosis (25, 26). While the production of viral molecules might result in the avoidance of the necroptotic process, it often causes inflammation and is one of the processes involved in combating certain viral infections. Necroptosis also promotes cancer progression or inhibition, transplant rejection, and ischemia-reperfusion damage (25, 26).

We investigated six necroptosis-associated lncRNAs to distinguish between cold and hot tumors in OC patients. Through the model, low-risk and high-risk groups of OC patients were separated, and several analyses were performed, including GSEA, Kaplan-Meier analysis, and drug-sensitive analyses. Although we found that risk groups might provide an insight into prognosis and systemic treatments, we could not distinguish hot tumors based on risk groups. In previous studies, molecular subtypes, also known as clusters, have been linked to tumor immunity suppression and microenvironment (27). Based on the expression of these lncRNAs, we grouped patients into two clusters. The immune microenvironments of the two clusters differed, as expected. There was an immunosuppressive TME in cluster 1.

Furthermore, cluster 2 had greater infiltration of CD8^+^ T cells, higher immune scores, and higher TIM3, PD-L1, and LAG3 levels, indicative of hot tumors (14). Necroptosis-associated lncRNAs might predict prognosis and guide individual therapy in OC patients. Moreover, necroptosis-associated IncRNAs might be used to differentiate between cold and hot tumors more accurately and rapidly (by performing imaging mass cytometry) than tumor biopsy (28).

The six lncRNAs, including AP003392.3, AL928654.1, AL133371.2, AC007991.4, AC011445.1, and LINC00996, were involved in the prognostic modeling of our study. The Sankey plot showed that these lncRNAs were associated with immunotherapy-related genes like USP22. AP003392.3 was associated with USP22. USP22 is a deubiquitinating enzyme that can affect tumor malignancy, metastasis, and prognosis (29). Targeting USP22 is a new strategy for potentiating anti-cancer immunity in PD-L1-amplified cancer (29, 30). AL928654.1 was associated with HSP90AA1. HSP90AA1 is an important molecular chaperon that is highly conserved throughout evolution. It is highly expressed when trauma, infection, and tumor stimulation are present. HSP90AA1 can enter the nucleus, stimulate immune memory formation, and participate in tumor development in the extracellular environment (31). AL928654.1 is a diagnostic and prognostic marker for hepatocellular carcinoma (32). AL133371.2, AC007991.4, and LINC00996 were relevant to TNFRSF1B. TNFRSF1B is more significantly upregulated in CD8+ T cells (33), which may also be relevant to immunotherapy. For treating autoimmune diseases, TNFRSF1B is a powerful therapeutic target (34). Recently, AC007991.4 was shown to be associated with the prognosis of gastric and ovarian cancer (35, 36), while LINC00996 can be used as a prognostic factor for colorectal and lung cancer (37, 38). There are no published studies related to AL133371.2. AC011445.1 is related to SIRT2 and SPATA2. SIRT2 is an NAD+-dependent deacetylase and is the only sirtuin protein found in the cytoplasm, mitochondria, and nucleus [36]. It can regulate autophagy in high-fat-exposed immune cells (39). Another member of the spermatogenesis-related protein family, SPATA2, is associated with the autoimmune disorder of psoriasis (40). The lncRNA AC011445.1 might be used as a prognostic marker for ovarian cancer (41, 42). Thus, necroptosis-associated lncRNAs are inextricably linked to immunotherapy.

We chose the SK-OV-3 cell line for this study partly because it is one of the most extensively used cell lines in ovarian cancer research. According to ATCC, it is resistant to cytotoxic chemicals, including diphtheria toxin and tumor necrosis factor, as well as, therapeutic anti-ovarian cancer medicines like cis-platinum and Adriamycin (43). The most extensively used normal ovarian epithelial cell line, Iose-80, served as the control group in this study.

Although our model was improved using various methods, there were still some limitations. We conducted a retrospective study, which might have introduced some bias in the results. Although checkpoint activation varied significantly among risk groups and clusters, we could not compare the corresponding checkpoint inhibitors, including PD-1 inhibitors, due to insufficient data on GDSC. Although we validated the expression of lncRNAs in the model with the ovarian cancer cell line SK-OV-3, we did not validate it with ovarian cancer tissue samples, making our results more conservative. Our results were partially consistent with those of TCGA and GETx, and further validation in future studies with more patients is required. The tests and the entire model sets were internally validated, but external validation of the prognosis was difficult. Although we retrieved all the information from GEO for the GDS3592, GSE54388, and GSE66957 series matrices, we were unable to obtain sufficient information for the lncRNAs. Due to the biases and limitations of commercial microarrays compared to the GTEx and TCGA data, we could not obtain appropriate information on lncRNAs. The cell heat map and the bubble plot of immune cells displayed results from multiple sources, which might be considered external verification. We aim to collect more clinical data to prove the importance of these necroptosis-associated long noncoding RNAs.

Cell death is also caused by necroptosis and lncRNAs. Through a caspase-independent mechanism, necroptosis can bypass apoptosis to cause cancer cell death (8). Apoptosis-related signaling pathways can be regulated by lncRNAs (44). Determining their interactions and mechanisms through experiments might provide an advanced strategy to kill cancer cells effectively while keeping healthy cells unharmed (6, 8). This treatment technique can lead to the advancement of immunotherapy and cancer research.

## Conclusions

Necroptosis-associated lncRNAs are a reliable predictor of prognosis and can provide a treatment strategy by identifying cold and hot tumor types. Their application might considerably improve individual therapy and the prognosis of patients. By focusing on necroptosis and lncRNAs, we might overcome systemic treatment failures and expand the field of immunotherapy. Consequently, the relationships among necroptosis, lncRNA, and OC, and the mechanism by which they are interrelated need to be further investigated.

## Data availability statement

Publicly available datasets were analyzed in this study. This data can be found here: GTEx (https://www.gtexportal.org/),TCGA (https://portal.gdc.cancer.gov/), GSEA (http://www.gsea-msigdb.org/gsea/index.jsp), GSVA (https://www.cancerrxgene.org/), and TIMER (http://timer.cistrome.org/).

## Author contributions

Y-BH and L-WF contributed equally to this work and should be considered co-first authors. Y-BH and L-WF conceived, designed, and write the manuscript. DH and S-LC performed data analysis. S-YS, K-LC and JM collected the data and arranged the figures. J-YL helped in the revise process. Y-LL performed RT-PCR experiments. H-PZ, LZ and Y-LL contributed to the project administration and edit the manuscript. All authors reviewed and approved the final manuscript.

## Funding

This work was supported by Research Projects of Zhejiang Chinese Medicine University (No. 2022JKZKTS26)(2022JKJNTZ16), Zhejiang young and middle-aged clinical famous Chinese medicine talents project (No. 1S22228) Hainan Province Health Industry Scientific Research Project (No. 21A200333), Sanya University and Medical Institutions Special Science and Technology Project (No. 2021GXYL29), Hainan Province Clinical Medical Center, Sanya Maternal and Child Health Hospital Golden Coconut Seeds (No. JYZZ-201903), Sanya Special Science and Technology Program for Universities and Medical Institutions (No. 2021GXYL29), Hainan Health Science Education Project (No. 21A200333).

## Acknowledgments

We sincerely thank Yu-long Dai from Tsinghua University School of Medicine, Ting-wenyi Hu from West China Hospital, Sichuan University and Qin-chao Ding from School of Medical Technology and Information Engineering, Zhejiang University of Traditional Chinese Medicine for their generous support of our study.

We also thank Shiyuan Tong from Fudan University, Haijing Fu and Huagen Li from Zhejiang University, Yuxuan Song from Peking University, and Ruifang Luo from Chongqing Medical University for solving the puzzles of our study.

## Conflict of interest

The authors declare that the research was conducted in the absence of any commercial or financial relationships that could be construed as a potential conflict of interest.

## Publisher’s note

All claims expressed in this article are solely those of the authors and do not necessarily represent those of their affiliated organizations, or those of the publisher, the editors and the reviewers. Any product that may be evaluated in this article, or claim that may be made by its manufacturer, is not guaranteed or endorsed by the publisher.

## Supplementary material

Supplementary Figure 1 (Image 1). The GSEA of the risk groups.

Supplementary Figure 2 (Image 2). **Supplementary material** for the risk model. **(A)** Correlation of risk scores with immune cells. **(B)** Drug sensitivity analysis in risk model.

Supplementary Figure 3 (Image 3). **Supplementary material** for the 2 clusters. **(A)** Heat map and CDF diagram. **(B)** Drug sensitivity analysis in 2 clusters. **(C)** Drug sensitivity analysis in 2 clusters.

Appendix D1 (Table 1): Correlation of necrotizing apoptosis-a genes with IncRNA

Appendix D2 (Table 2): The data of immune infiltrating cell from different platforms in risk model

Appendix D3 (Table 3): The data of immune infiltrating cell from different platforms in cluster

Appendix T1 (Table 4): Necroptosis-Associated gene catalog and references

## Abbreviations

OC, Ovarian Cancer; TCGA, The Cancer Genome Atlas; GTEx, Genotype-Tissue Expression Project; LincRNAs, Long Noncoding RNAs; LASSO, least absolute shrinkage and selection operator; ICB, Immune checkpoint blockade; GSEA, Gene Set Enrichment Analysis; GDSC, Genomics of Drug Sensitivity in Cancer; RS, Risk score; OS, Overall survival; IC50, The half-maximal inhibitory concentration; T-SNE,T-distributed stochastic neighbor embedding,; PCA, Principal component analysis; ROC, Receiver operating characteristics; AUC, The area under the ROC curve; KEGG, Kyoto Encyclopedia of Genes and Genomes.
