# Supplementary figures and images for: Necroptosis-associated long noncoding RNAs can predict prognosis and differentiate between cold and hot tumors in ovarian cancer
Source: Front Oncol. 2022 Jul 28;12:967207. doi: 10.3389/fonc.2022.967207 (PMC9366220; doi:10.3389/fonc.2022.967207)

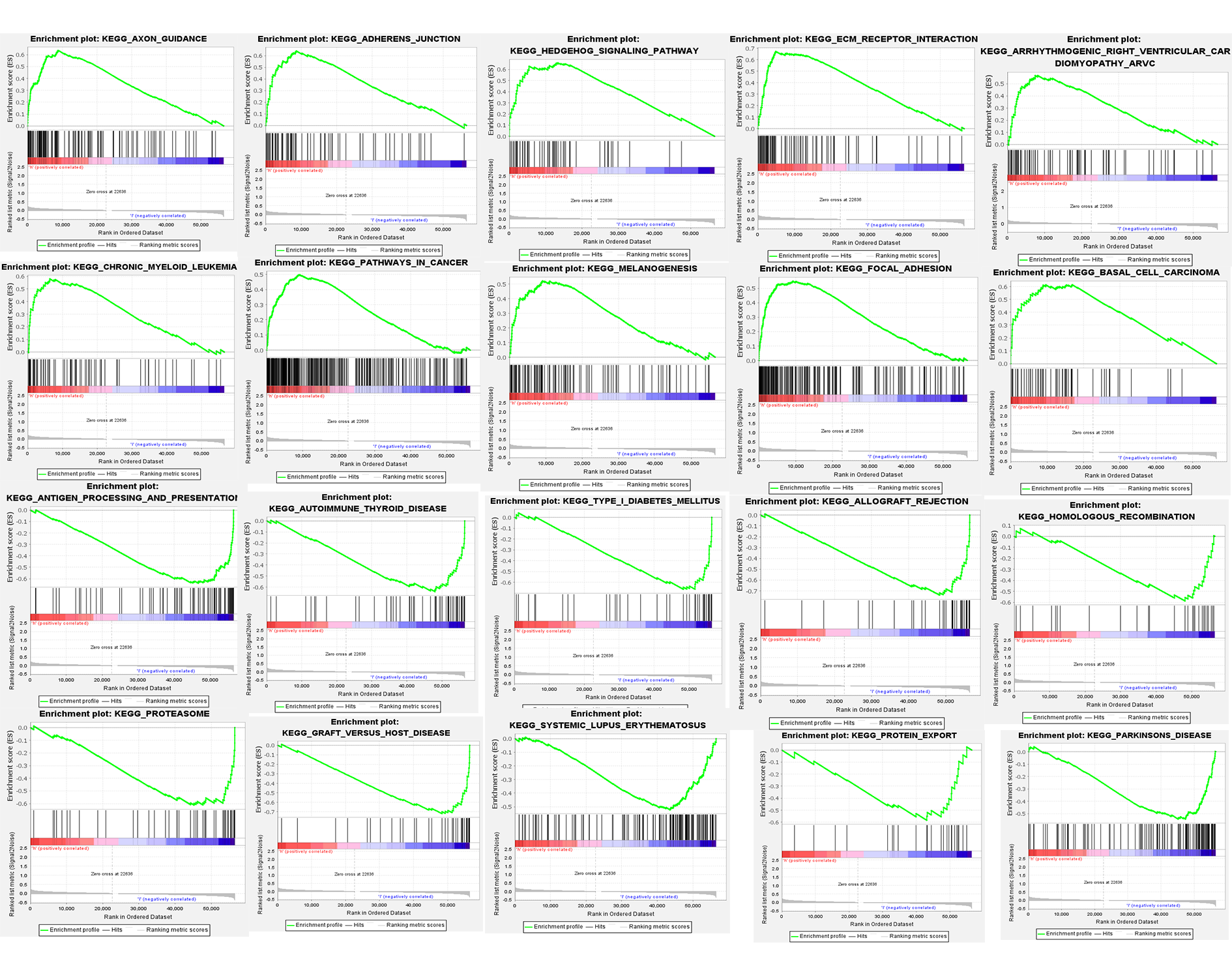

Supplement: Supplementary Figure 1 — (Image 1). The GSEA of the risk groups. [file Image_1.tif]

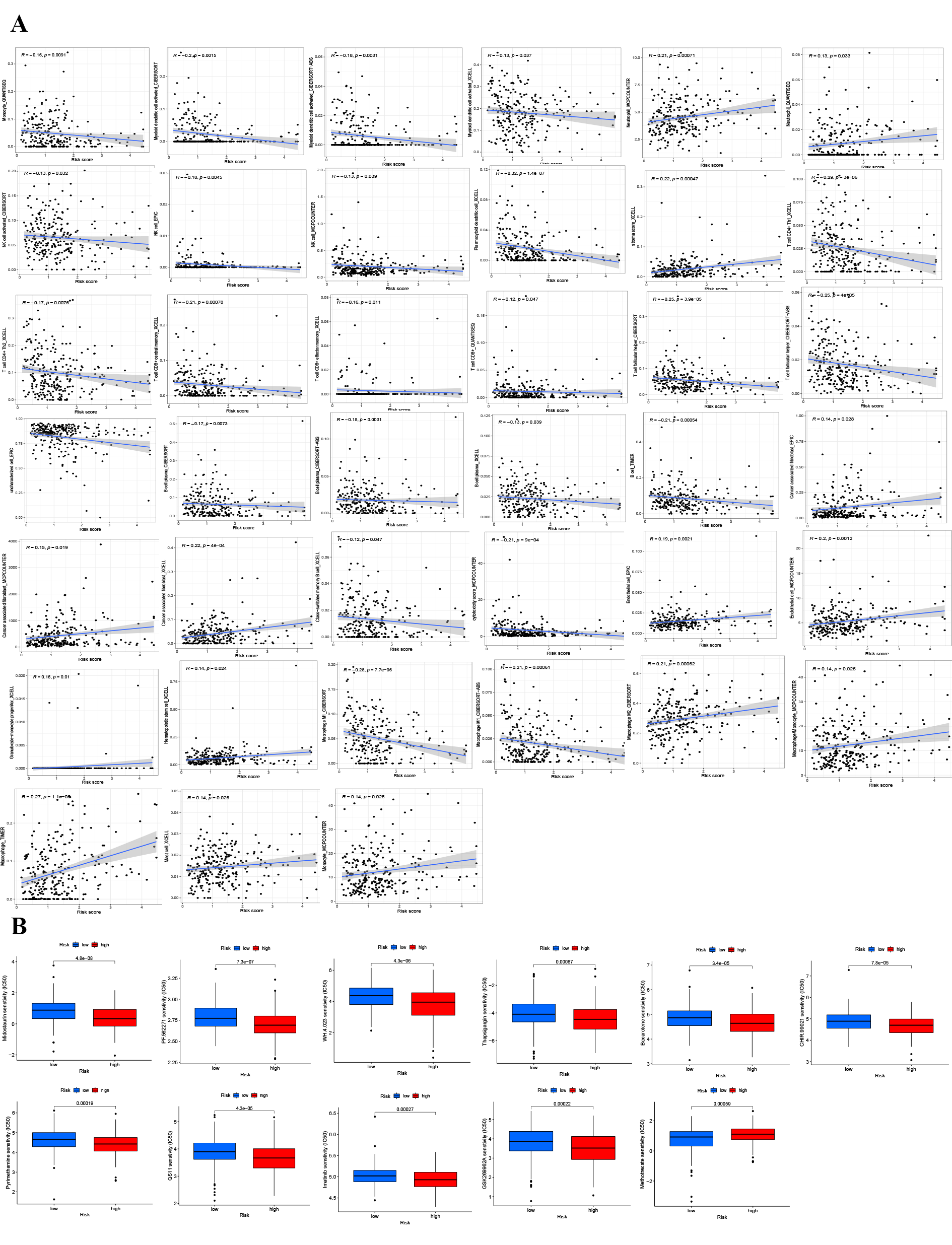

Supplement: Supplementary Figure 2 — (Image 2). Supplementary material for the risk model. (A) Correlation of risk scores with immune cells. (B) Drug sensitivity analysis in risk model. [file Image_2.tif]

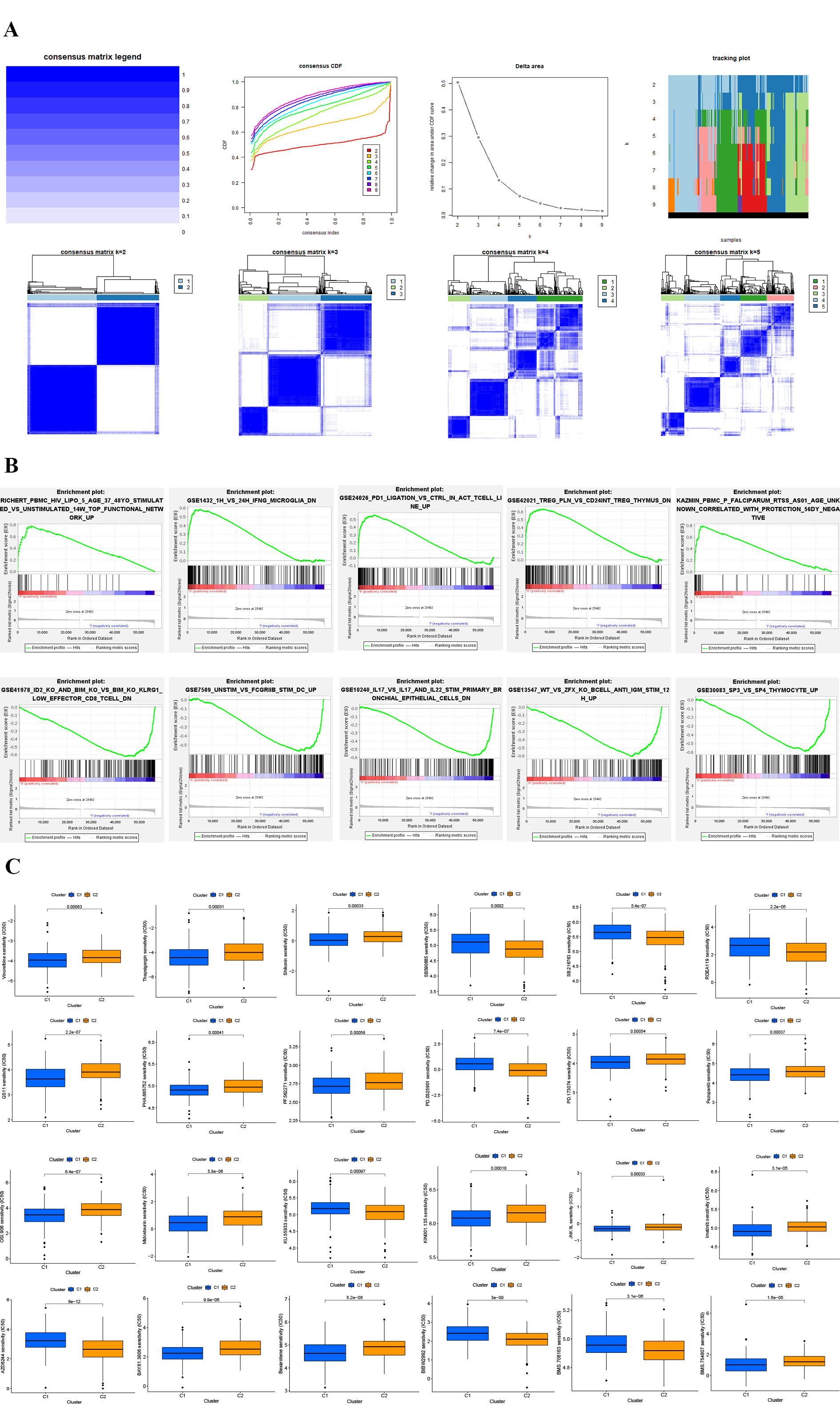

Supplement: Supplementary Figure 3 — (Image 3). Supplementary material for the 2 clusters. (A) Heat map and CDF diagram. (B) Drug sensitivity analysis in 2 clusters. (C) Drug sensitivity analysis in 2 clusters. [file Image_3.tif]
