# Supplementary material for: Necroptosis-associated long noncoding RNAs can predict prognosis and differentiate between cold and hot tumors in ovarian cancer
Source: Front Oncol. 2022 Jul 28;12:967207. doi: 10.3389/fonc.2022.967207 (PMC9366220; doi:10.3389/fonc.2022.967207)
Supplement: Appendix D1 — (Table 1): Correlation of necrotizing apoptosis-a genes with IncRNA [file Table_1.docx]

| NRG | lncRNA | cor | pvalue | Regulation | |
| --- | --- | --- | --- | --- | --- |
| PLK1 | LINC01224 | 0.435494856998967 | 5.67468063678745e-19 | | postive |
| AXL | AP000892.3 | 0.401295387179174 | 4.26247382000218e-16 | | postive |
| CFLAR | HCG27 | 0.427587650762889 | 2.80702760879532e-18 | | postive |
| FASLG | LINC02384 | 0.413840198352803 | 4.09970758583396e-17 | | postive |
| MLKL | AL022316.1 | 0.471178029787738 | 2.42719076196652e-22 | | postive |
| FASLG | LINC02195 | 0.650223929901114 | 6.45852694183895e-47 | | postive |
| MLKL | LINC02195 | 0.441056966140717 | 1.79698246830472e-19 | | postive |
| TARDBP | AL513477.2 | 0.457249465808531 | 5.58966684329066e-21 | | postive |
| STAT3 | MYCNOS | -0.431745235354003 | 1.21745495257492e-18 | | negative |
| MYCN | MYCNOS | 0.799886404353409 | 1.3006166085059e-85 | | postive |
| AXL | AC087286.2 | 0.441813352243987 | 1.53433142954501e-19 | | postive |
| TSC1 | AC008608.2 | -0.41365076597633 | 4.2503918513767e-17 | | negative |
| USP22 | AC008608.2 | -0.436022285953059 | 5.09306530633788e-19 | | negative |
| MPG | AC008608.2 | 0.424203815007377 | 5.49347639920759e-18 | | postive |
| CDKN2A | CDKN2A-DT | 0.625654758395413 | 1.4323626599033e-42 | | postive |
| TARDBP | AC024267.3 | 0.406631377983253 | 1.59347656705188e-16 | | postive |
| FAS | LINC01615 | 0.401548269629941 | 4.06989581467743e-16 | | postive |
| STAT3 | AC124016.1 | -0.449035566652969 | 3.32634738404066e-20 | | negative |
| FASLG | USP30-AS1 | 0.570305043356307 | 4.45374719480619e-34 | | postive |
| MLKL | USP30-AS1 | 0.585714239158828 | 2.79948407655623e-36 | | postive |
| TLR3 | USP30-AS1 | 0.554181725766586 | 6.78621927547149e-32 | | postive |
| ZBP1 | USP30-AS1 | 0.593880083078361 | 1.70579380333139e-37 | | postive |
| TNFSF10 | USP30-AS1 | 0.525109411334985 | 3.02271517391372e-28 | | postive |
| CYLD | USP30-AS1 | 0.449474522778066 | 3.02762031919446e-20 | | postive |
| USP22 | USP30-AS1 | -0.429258274808572 | 2.00940802230177e-18 | | negative |
| DDX58 | USP30-AS1 | 0.448646152997258 | 3.61553021385911e-20 | | postive |
| USP22 | AC023644.1 | -0.412180522471389 | 5.62028977470245e-17 | | negative |
| USP22 | DLG3-AS1 | -0.433407487201746 | 8.68956346058328e-19 | | negative |
| APP | DLG3-AS1 | -0.403344278445784 | 2.9274525863258e-16 | | negative |
| TNFRSF1B | AC068580.1 | 0.460666593486913 | 2.6237748063514e-21 | | postive |
| AXL | HECW2-AS1 | 0.448886465807471 | 3.43427628440066e-20 | | postive |
| AXL | CLMAT3 | 0.440022358581519 | 2.22911974046923e-19 | | postive |
| USP22 | AC010913.1 | -0.432425834981194 | 1.06068221162618e-18 | | negative |
| FASLG | AF127936.1 | 0.515994026779043 | 3.57743514426055e-27 | | postive |
| MLKL | AF127936.1 | 0.519596117207977 | 1.3594337990086e-27 | | postive |
| TLR3 | AF127936.1 | 0.480045296575857 | 3.0534482296271e-23 | | postive |
| ZBP1 | AF127936.1 | 0.589786815510171 | 7.0041767656966e-37 | | postive |
| TNFRSF1B | AF127936.1 | 0.518789662681807 | 1.68994830441416e-27 | | postive |
| CYLD | AF127936.1 | 0.418571454563554 | 1.65192629817852e-17 | | postive |
| DDX58 | AF127936.1 | 0.448232424244956 | 3.94990009638057e-20 | | postive |
| TLR3 | AL357054.4 | 0.486491185772643 | 6.51127628539903e-24 | | postive |
| ZBP1 | AL357054.4 | 0.440743830815915 | 1.91824254876405e-19 | | postive |
| CYLD | AL357054.4 | 0.429921506204838 | 1.75877052242277e-18 | | postive |
| FASLG | AC004921.1 | 0.446823942246579 | 5.33297368210185e-20 | | postive |
| DNMT1 | AC114271.1 | 0.525178991754351 | 2.96537767998336e-28 | | postive |
| TARDBP | AC009095.1 | 0.402374068330004 | 3.49862147380673e-16 | | postive |
| TARDBP | AP001628.1 | 0.42245702302599 | 7.74634679961679e-18 | | postive |
| SQSTM1 | MIR181A2HG | -0.448103711977535 | 4.06000133616412e-20 | | negative |
| TSC1 | SLC25A25-AS1 | 0.472702306539848 | 1.70687334394816e-22 | | postive |
| MAP3K7 | TRAF3IP2-AS1 | 0.451602131015549 | 1.91506818258149e-20 | | postive |
| ATRX | AC004000.1 | 0.43707393937031 | 4.10294003390966e-19 | | postive |
| TSC1 | YEATS2-AS1 | 0.435582839042705 | 5.57331020783253e-19 | | postive |
| MPG | YEATS2-AS1 | -0.455735904339686 | 7.7928395590312e-21 | | negative |
| TARDBP | YEATS2-AS1 | 0.439165432394902 | 2.66322671524466e-19 | | postive |
| RNF31 | AC026726.1 | -0.492001613454313 | 1.69261901561591e-24 | | negative |
| MPG | KCNQ1OT1 | -0.407256536090626 | 1.41833274332938e-16 | | negative |
| TARDBP | KCNQ1OT1 | 0.459853632909109 | 3.14343554066733e-21 | | postive |
| FASLG | LINC00426 | 0.772461644226091 | 2.43424248991714e-76 | | postive |
| MLKL | LINC00426 | 0.517110634227069 | 2.65366168054229e-27 | | postive |
| TNFRSF1B | LINC00426 | 0.432739743016799 | 9.95239111771333e-19 | | postive |
| GATA3 | LINC00426 | 0.412349544544413 | 5.44303039708461e-17 | | postive |
| MLKL | AC245128.3 | 0.416765551576855 | 2.34102769494984e-17 | | postive |
| TNF | AC245128.3 | 0.486769497370322 | 6.08650408793881e-24 | | postive |
| TNFRSF1B | AC245128.3 | 0.482560004389437 | 1.67747887740275e-23 | | postive |
| SIRT2 | AC022144.1 | 0.400915617704936 | 4.56859461844556e-16 | | postive |
| USP22 | AP003392.3 | -0.407738340950918 | 1.2963837222425e-16 | | negative |
| RNF31 | AP003392.3 | -0.434627307470347 | 6.77656690308371e-19 | | negative |
| TARDBP | AC020915.2 | 0.438221498721965 | 3.23799299908299e-19 | | postive |
| TARDBP | AC018690.1 | 0.409140417376912 | 9.97130519491928e-17 | | postive |
| FASLG | AC146944.2 | 0.472479229250583 | 1.79732406078229e-22 | | postive |
| MLKL | AC146944.2 | 0.418225610377181 | 1.76627636284295e-17 | | postive |
| TNFRSF1B | AC146944.2 | 0.421213604497362 | 9.88120083360278e-18 | | postive |
| TARDBP | AC146944.2 | -0.412961576029961 | 4.84593652814105e-17 | | negative |
| FASLG | AC138207.5 | 0.439505460433217 | 2.48182834329395e-19 | | postive |
| MLKL | AC138207.5 | 0.487580646424991 | 4.99832202792043e-24 | | postive |
| TNFRSF1B | AC138207.5 | 0.54694818689785 | 5.92852843626721e-31 | | postive |
| TARDBP | AC005546.1 | 0.402115966388474 | 3.66814715778597e-16 | | postive |
| USP22 | LINC00853 | -0.407890013748111 | 1.26017022307659e-16 | | negative |
| USP22 | AC010531.6 | -0.40048337257709 | 4.94331906361616e-16 | | negative |
| TARDBP | MCCC1-AS1 | 0.452342014924545 | 1.6318589582842e-20 | | postive |
| STUB1 | ZNF213-AS1 | 0.450335926145321 | 2.51613491053583e-20 | | postive |
| BACH2 | LINC01801 | 0.420264848286465 | 1.18898652835882e-17 | | postive |
| FASLG | AC006033.2 | 0.633097362055068 | 7.60191741540352e-44 | | postive |
| MLKL | AC006033.2 | 0.527621425467426 | 1.5097523226237e-28 | | postive |
| TNFSF10 | AC006033.2 | 0.409519452758789 | 9.28637486486798e-17 | | postive |
| TNFRSF1B | AC006033.2 | 0.558265295024071 | 1.94982599298371e-32 | | postive |
| CYLD | AC006033.2 | 0.423471795622449 | 6.34597278608307e-18 | | postive |
| AXL | AC006033.2 | 0.405248703210627 | 2.05973033432953e-16 | | postive |
| STAT3 | AL133260.1 | -0.427183466078168 | 3.04263473771041e-18 | | negative |
| SLC39A7 | AL133260.1 | -0.4527699374014 | 1.48733219952431e-20 | | negative |
| TARDBP | AC068790.7 | 0.464152758375445 | 1.20227989156261e-21 | | postive |
| PLK1 | TMPO-AS1 | 0.452476499168102 | 1.58500824751796e-20 | | postive |
| CFLAR | AC055855.1 | 0.431939803957684 | 1.17044723347843e-18 | | postive |
| MLKL | AC110995.1 | 0.412885665616095 | 4.9163442086149e-17 | | postive |
| TNFRSF1B | AC110995.1 | 0.542253598166277 | 2.35362694439571e-30 | | postive |
| AXL | AC110995.1 | 0.432347322343506 | 1.07770125814239e-18 | | postive |
| PANX1 | AC108673.3 | -0.489740428562939 | 2.95079270530537e-24 | | negative |
| RNF31 | AC108673.3 | -0.459393623150995 | 3.48111264257272e-21 | | negative |
| CFLAR | SH3BP5-AS1 | 0.425962902577341 | 3.87853765551828e-18 | | postive |
| FASLG | LINC01150 | 0.466405664384944 | 7.22612389972527e-22 | | postive |
| MLKL | LINC01150 | 0.401790622159349 | 3.89336254218557e-16 | | postive |
| TNFRSF1B | LINC01150 | 0.5654270030999 | 2.09803313747492e-33 | | postive |
| AXL | LINC01150 | 0.447848109721136 | 4.28767880588801e-20 | | postive |
| TRIM11 | AL670729.1 | 0.708735148286526 | 4.35876261452097e-59 | | postive |
| TARDBP | AC024075.3 | 0.40656187935238 | 1.61421249096123e-16 | | postive |
| TSC1 | NCK1-DT | -0.410961126013643 | 7.07840729340686e-17 | | negative |
| RNF31 | COA6-AS1 | -0.41033085823959 | 7.97174874876155e-17 | | negative |
| FASLG | MMP2-AS1 | 0.525222042913191 | 2.93044101255639e-28 | | postive |
| MLKL | MMP2-AS1 | 0.473826484175034 | 1.31504820500023e-22 | | postive |
| TNFRSF1B | MMP2-AS1 | 0.427225744726474 | 3.01710629658211e-18 | | postive |
| AXL | MSC-AS1 | 0.437472300294882 | 3.77960977577166e-19 | | postive |
| CFLAR | INE1 | 0.417069744065742 | 2.20782896174284e-17 | | postive |
| USP22 | SPINT1-AS1 | -0.481689078582023 | 2.06534772666401e-23 | | negative |
| TSC1 | AC000120.1 | 0.41349788743465 | 4.37595532518554e-17 | | postive |
| MAP3K7 | LINC02428 | 0.43504082533387 | 6.22734175270563e-19 | | postive |
| TARDBP | AL031714.1 | 0.46436699435535 | 1.14564704837229e-21 | | postive |
| TLR3 | LINC01143 | -0.425026941972439 | 4.66891836429444e-18 | | negative |
| USP22 | AC005288.1 | 0.42131863094326 | 9.68049401526479e-18 | | postive |
| STAT3 | AC005288.1 | 0.50659518948124 | 4.23402741730692e-26 | | postive |
| TARDBP | AC080162.1 | 0.41156227135245 | 6.31830965123926e-17 | | postive |
| AXL | AC080038.1 | 0.482951724467976 | 1.52736151516842e-23 | | postive |
| AXL | AC009093.1 | 0.485226742497475 | 8.83972465830047e-24 | | postive |
| USP22 | AC108449.2 | 0.468705809717312 | 4.2802968727825e-22 | | postive |
| APP | AC108449.2 | 0.430539564199185 | 1.55301742594858e-18 | | postive |
| TARDBP | GAPLINC | -0.449033143332686 | 3.32807454938418e-20 | | negative |
| CFLAR | AC105020.1 | 0.437689259353451 | 3.61419960572463e-19 | | postive |
| USP22 | AC138696.2 | -0.436655513139305 | 4.47186211688237e-19 | | negative |
| AXL | AL133415.1 | 0.427702268791228 | 2.74354442753067e-18 | | postive |
| DNMT1 | AL807752.5 | 0.405167028033348 | 2.09111750306002e-16 | | postive |
| MPG | AL807752.5 | -0.406213827067533 | 1.7221115132459e-16 | | negative |
| FASLG | U62317.1 | 0.494113989834923 | 1.00331592625891e-24 | | postive |
| MLKL | U62317.1 | 0.535121995922639 | 1.83465646356778e-29 | | postive |
| ZBP1 | U62317.1 | 0.521864172187008 | 7.34831302249818e-28 | | postive |
| TNFSF10 | U62317.1 | 0.451230296641021 | 2.07514720524702e-20 | | postive |
| TNFRSF1B | U62317.1 | 0.403611577255169 | 2.78688640765692e-16 | | postive |
| CASP8 | AC104667.2 | 0.418059906597687 | 1.82378683168905e-17 | | postive |
| IDH2 | IDH2-DT | 0.462569787417881 | 1.71546477160722e-21 | | postive |
| FASLG | AC004687.1 | 0.581323471215778 | 1.2200307280329e-35 | | postive |
| MLKL | AC004687.1 | 0.55204431652024 | 1.29468642116658e-31 | | postive |
| TNFRSF1B | AC004687.1 | 0.579842189791461 | 1.99465052603272e-35 | | postive |
| MAP3K7 | AL133338.1 | 0.537354884265247 | 9.69639309276608e-30 | | postive |
| FASLG | TRBV11-2 | 0.489952579619562 | 2.80135382609563e-24 | | postive |
| ATRX | FTX | 0.435331515561646 | 5.8677107447366e-19 | | postive |
| CFLAR | AL691482.3 | 0.420719660599891 | 1.08813408657227e-17 | | postive |
| TARDBP | BACE1-AS | 0.401709316908413 | 3.95173204741575e-16 | | postive |
| BACH2 | NUP50-DT | -0.418116523077287 | 1.80393307127096e-17 | | negative |
| TSC1 | AC129510.1 | 0.416123141006538 | 2.64880312852371e-17 | | postive |
| SIRT3 | AC136475.1 | 0.427437429162767 | 2.89242187811156e-18 | | postive |
| TSC1 | N4BP2L2-IT2 | 0.417547692019627 | 2.01344624944022e-17 | | postive |
| RNF31 | AC138932.1 | 0.487301704688305 | 5.34893260279177e-24 | | postive |
| TARDBP | AL355488.1 | 0.499247637857535 | 2.77234394915041e-25 | | postive |
| FASLG | AL590764.1 | 0.640057390472974 | 4.53446819082123e-45 | | postive |
| MLKL | AL590764.1 | 0.525819928860492 | 2.48528357569926e-28 | | postive |
| TNFRSF1B | AL590764.1 | 0.506672552196802 | 4.15006987953763e-26 | | postive |
| TARDBP | AL590764.1 | -0.459573866191443 | 3.34474450143761e-21 | | negative |
| TARDBP | AC007938.3 | 0.415218696298439 | 3.15049312336598e-17 | | postive |
| TNFRSF1B | AC068580.3 | 0.488114738258416 | 4.38908843914901e-24 | | postive |
| TARDBP | PTOV1-AS2 | 0.437184275289755 | 4.01075580077294e-19 | | postive |
| STUB1 | AC092117.1 | 0.423422141565844 | 6.40829352297503e-18 | | postive |
| TNFRSF1B | AC005076.1 | -0.457558242239024 | 5.22224688715181e-21 | | negative |
| STAT3 | AC005076.1 | -0.407519008389802 | 1.35056565264945e-16 | | negative |
| TSC1 | AC005104.1 | 0.403167768669334 | 3.02406643086722e-16 | | postive |
| CFLAR | AC005104.1 | 0.401420500444638 | 4.16610379364115e-16 | | postive |
| MPG | AL355001.2 | 0.466292983812043 | 7.41315041633293e-22 | | postive |
| TSC1 | AL161452.1 | 0.413312702511124 | 4.53294080895178e-17 | | postive |
| TRAF2 | AL161452.1 | 0.477363894666957 | 5.75189008173812e-23 | | postive |
| RNF31 | AC019131.2 | -0.436389492862457 | 4.72318290971129e-19 | | negative |
| FASLG | AP002954.1 | 0.474937822362267 | 1.01522834658618e-22 | | postive |
| MLKL | AP002954.1 | 0.493003977013733 | 1.32124195141967e-24 | | postive |
| TNFRSF1B | AP002954.1 | 0.434961210777687 | 6.32956419369245e-19 | | postive |
| RIPK1 | GMDS-DT | 0.534664283265975 | 2.08963479924243e-29 | | postive |
| TNFRSF1B | AP002360.1 | -0.433218892846897 | 9.02931746216329e-19 | | negative |
| FASLG | AC005515.1 | 0.438299356612863 | 3.18629388507445e-19 | | postive |
| MLKL | AC005515.1 | 0.466079298491157 | 7.78101846662098e-22 | | postive |
| TLR3 | AC005515.1 | 0.415601266067138 | 2.92781122859191e-17 | | postive |
| ZBP1 | AC005515.1 | 0.672968289785331 | 2.58069163948866e-51 | | postive |
| TNFSF10 | AC005515.1 | 0.458530773498579 | 4.21363788264649e-21 | | postive |
| DDX58 | AC005515.1 | 0.509310294067146 | 2.09001593383957e-26 | | postive |
| HSP90AA1 | AL928654.1 | 0.425249727107999 | 4.46751721804387e-18 | | postive |
| MAP3K7 | AC019205.1 | 0.453903298873799 | 1.1626931864657e-20 | | postive |
| TARDBP | ANKRD10-IT1 | 0.524714924848596 | 3.36911159785845e-28 | | postive |
| CFLAR | AC139795.2 | 0.431704195542825 | 1.22760478100888e-18 | | postive |
| TARDBP | AC139795.2 | 0.410605164888091 | 7.5701009144531e-17 | | postive |
| STAT3 | AC007563.3 | -0.403239708261667 | 2.98431765295045e-16 | | negative |
| TNFRSF1B | AC106739.1 | 0.449147772570978 | 3.24733442011492e-20 | | postive |
| RNF31 | AP001094.1 | -0.408210045868631 | 1.18698779346886e-16 | | negative |
| ATRX | AC006141.1 | 0.433042955993142 | 9.35803301887573e-19 | | postive |
| TNFRSF1A | AC022509.3 | 0.45625791472625 | 6.95034056917471e-21 | | postive |
| RNF31 | AC107027.1 | -0.425697349108429 | 4.08833876029747e-18 | | negative |
| USP22 | OSMR-AS1 | -0.423442849366532 | 6.38223039048341e-18 | | negative |
| ZBP1 | AC020931.1 | 0.434609134540518 | 6.8017669384979e-19 | | postive |
| DNMT1 | AC020931.1 | 0.431397920709307 | 1.30602574566622e-18 | | postive |
| DDX58 | AC020931.1 | 0.443150030422134 | 1.15938146218973e-19 | | postive |
| TARDBP | AC022092.1 | -0.411716314843827 | 6.1368132261325e-17 | | negative |
| TLR3 | AC007541.1 | -0.409674246530204 | 9.02014105004421e-17 | | negative |
| TARDBP | AC006435.2 | 0.417233161451094 | 2.13937711908089e-17 | | postive |
| TARDBP | AC005785.1 | 0.513734490034812 | 6.52561367143796e-27 | | postive |
| TARDBP | AL512413.1 | 0.407995957151114 | 1.23546631001048e-16 | | postive |
| MLKL | NRIR | 0.430173568205966 | 1.67180796568395e-18 | | postive |
| TLR3 | NRIR | 0.419835937015027 | 1.29248587861211e-17 | | postive |
| ZBP1 | NRIR | 0.62862821971998 | 4.47473334076746e-43 | | postive |
| TNFSF10 | NRIR | 0.482106686598555 | 1.86943065105118e-23 | | postive |
| DDX58 | NRIR | 0.659466833172844 | 1.17203021398573e-48 | | postive |
| AXL | AC112721.2 | 0.415883598031879 | 2.77346545952235e-17 | | postive |
| TARDBP | AC012467.2 | 0.403210968409943 | 3.0001353779471e-16 | | postive |
| MLKL | AC018755.4 | 0.456054269398148 | 7.2677677189271e-21 | | postive |
| TNFRSF1B | AC018755.4 | 0.412376045507901 | 5.41574055351314e-17 | | postive |
| ITPK1 | AL049840.4 | 0.455054486347266 | 9.04546508859864e-21 | | postive |
| SIRT1 | ZNF32-AS2 | 0.415135409512797 | 3.20113385753383e-17 | | postive |
| SIRT3 | CD81-AS1 | 0.454595749673079 | 9.99837637678562e-21 | | postive |
| TARDBP | LINC01772 | 0.464148758985783 | 1.20336293199085e-21 | | postive |
| ZBP1 | AC116407.2 | 0.489755175067852 | 2.94015570615648e-24 | | postive |
| DDX58 | AC116407.2 | 0.459092601464551 | 3.72117187509812e-21 | | postive |
| USP22 | MIR200CHG | -0.425593624366531 | 4.17328299962509e-18 | | negative |
| TSC1 | CTBP1-AS | 0.45797903409312 | 4.75957774581166e-21 | | postive |
| MLKL | AC124319.1 | 0.542576648286769 | 2.14207189134789e-30 | | postive |
| TLR3 | AC124319.1 | 0.498982792786085 | 2.96412340881398e-25 | | postive |
| ZBP1 | AC124319.1 | 0.532842851966226 | 3.50039861614416e-29 | | postive |
| TNFSF10 | AC124319.1 | 0.466346707641519 | 7.32339237974062e-22 | | postive |
| TNFRSF1B | AC124319.1 | 0.488408687044163 | 4.08567321528498e-24 | | postive |
| CYLD | AC124319.1 | 0.45644370144549 | 6.67268500697746e-21 | | postive |
| STAT3 | AC124319.1 | 0.449336382288467 | 3.11866316652819e-20 | | postive |
| CFLAR | AC124319.1 | 0.44086950968191 | 1.86863597624537e-19 | | postive |
| DDX58 | AC124319.1 | 0.532427216657302 | 3.93598024916671e-29 | | postive |
| USP22 | ARHGAP26-AS1 | -0.499004695975049 | 2.94777831896638e-25 | | negative |
| APP | ARHGAP26-AS1 | -0.434888889638563 | 6.42385196158151e-19 | | negative |
| TNFSF10 | AC008760.2 | 0.422078651552653 | 8.34279594331399e-18 | | postive |
| CYLD | SNHG11 | -0.478547430448977 | 4.35230099585588e-23 | | negative |
| KLF9 | AC129492.1 | 0.458643917786438 | 4.10955618197129e-21 | | postive |
| CFLAR | AC137932.3 | 0.402065690509763 | 3.70209518666721e-16 | | postive |
| FASLG | AC011899.3 | 0.420612306203985 | 1.11115326057856e-17 | | postive |
| TNFRSF1B | AC011899.3 | 0.407181496589104 | 1.43831332971129e-16 | | postive |
| USP22 | AC073046.1 | 0.523491688709107 | 4.71226143780748e-28 | | postive |
| RNF31 | AC073046.1 | 0.467022627269247 | 6.28160419639424e-22 | | postive |
| APP | AC073046.1 | 0.406861034061942 | 1.52679836558705e-16 | | postive |
| USP22 | AP003352.1 | -0.432312629873106 | 1.08530678544376e-18 | | negative |
| MLKL | TNKS2-AS1 | -0.444785824266648 | 8.21427064403233e-20 | | negative |
| STAT3 | TNKS2-AS1 | -0.408164848210141 | 1.19706430381414e-16 | | negative |
| FASLG | AL591468.1 | 0.617301131011034 | 3.52211484775503e-41 | | postive |
| MLKL | AL591468.1 | 0.524139390695731 | 3.94591454409892e-28 | | postive |
| ZBP1 | AL591468.1 | 0.413595309698839 | 4.29552510545557e-17 | | postive |
| TNFRSF1B | AL591468.1 | 0.442397142468638 | 1.35780145454442e-19 | | postive |
| RNF31 | AC073896.4 | -0.418935289668225 | 1.53948098545681e-17 | | negative |
| DDX58 | EBLN3P | 0.415946096019067 | 2.74039536111924e-17 | | postive |
| TARDBP | AC073869.1 | 0.513076463065697 | 7.76745786056432e-27 | | postive |
| DNMT1 | UBE2D3-AS1 | -0.400384306095344 | 5.03336220154343e-16 | | negative |
| TARDBP | AL139287.1 | 0.618521845001982 | 2.2191118319352e-41 | | postive |
| PLK1 | AC099850.3 | 0.562589632614901 | 5.10752226213238e-33 | | postive |
| TARDBP | AC002398.1 | 0.42897755587848 | 2.12578405656981e-18 | | postive |
| CFLAR | AP4B1-AS1 | 0.41277735913713 | 5.01854236585179e-17 | | postive |
| SIRT3 | AC136475.2 | 0.421289114742543 | 9.73649141963529e-18 | | postive |
| FASLG | LINC01871 | 0.762274197907352 | 3.20468922714389e-73 | | postive |
| MLKL | LINC01871 | 0.558578621220887 | 1.77062883761092e-32 | | postive |
| RIPK3 | LINC01871 | 0.423048555652315 | 6.89687328995351e-18 | | postive |
| TLR3 | LINC01871 | 0.410164790603691 | 8.22500498284012e-17 | | postive |
| TNFSF10 | LINC01871 | 0.412699074062057 | 5.09370885073805e-17 | | postive |
| TNFRSF1B | LINC01871 | 0.447841991260621 | 4.29328009269925e-20 | | postive |
| PLK1 | TYMSOS | 0.415269433434947 | 3.12003019112328e-17 | | postive |
| TNF | AC092484.1 | 0.409816828994916 | 8.78155085579409e-17 | | postive |
| TARDBP | AC004951.1 | 0.404703640505511 | 2.27828751632146e-16 | | postive |
| PANX1 | AC048341.2 | -0.408277153088271 | 1.17218017141368e-16 | | negative |
| TNFSF10 | LINC02068 | 0.4301679538439 | 1.67369808985559e-18 | | postive |
| BCL2 | AC027097.2 | 0.445262829533741 | 7.42635716928124e-20 | | postive |
| RNF31 | AC008035.1 | -0.403082171480072 | 3.07203892051778e-16 | | negative |
| FASLG | LINC02362 | 0.437568540656117 | 3.70533632812778e-19 | | postive |
| TARDBP | AC095057.3 | 0.48716596878418 | 5.52821059529645e-24 | | postive |
| PLK1 | ATP2A1-AS1 | 0.403712620831546 | 2.73549420685105e-16 | | postive |
| TARDBP | AC010834.3 | 0.426509140375755 | 3.47971075967776e-18 | | postive |
| FASLG | LINC00861 | 0.625022674481822 | 1.83130913743096e-42 | | postive |
| MLKL | LINC00861 | 0.47934446743763 | 3.605002953416e-23 | | postive |
| TNFRSF1B | LINC00861 | 0.456896105452722 | 6.04145088632548e-21 | | postive |
| RNF31 | AC135507.1 | -0.425787207425698 | 4.01612624993506e-18 | | negative |
| TARDBP | AC068888.2 | 0.411091184040034 | 6.90669695195128e-17 | | postive |
| FASLG | AC007728.2 | 0.60534571669384 | 2.92459368425406e-39 | | postive |
| MLKL | AC007728.2 | 0.61687438290494 | 4.13744450539761e-41 | | postive |
| TLR3 | AC007728.2 | 0.464127664731459 | 1.20909121344629e-21 | | postive |
| ZBP1 | AC007728.2 | 0.498024166108429 | 3.7742482340294e-25 | | postive |
| TNFSF10 | AC007728.2 | 0.467620294819154 | 5.48302958361863e-22 | | postive |
| TNFRSF1B | AC007728.2 | 0.459022358415641 | 3.77949496459195e-21 | | postive |
| CYLD | AC007728.2 | 0.683884494826085 | 1.43881566580439e-53 | | postive |
| DDX58 | AC007728.2 | 0.438474402014515 | 3.07300802291396e-19 | | postive |
| TLR3 | AC009950.1 | 0.416664964470832 | 2.38678591196128e-17 | | postive |
| ZBP1 | AC009950.1 | 0.605087904671972 | 3.21041482427384e-39 | | postive |
| DDX58 | AC009950.1 | 0.658372685745599 | 1.89788423440522e-48 | | postive |
| CFLAR | PSMD6-AS2 | 0.433219393667029 | 9.02839815194835e-19 | | postive |
| TARDBP | AC109992.2 | 0.404006455687304 | 2.59127457732972e-16 | | postive |
| TARDBP | AC020612.3 | 0.45161279112921 | 1.91066292414623e-20 | | postive |
| TRIM11 | OBSCN-AS1 | 0.409014233654827 | 1.02101486764336e-16 | | postive |
| AXL | ACTA2-AS1 | 0.502484581961833 | 1.21829578688362e-25 | | postive |
| TARDBP | LINC01355 | 0.592855477276593 | 2.43388490609694e-37 | | postive |
| CFLAR | AC006480.2 | 0.418545309570363 | 1.66031048197071e-17 | | postive |
| MLKL | AL133371.2 | 0.41186237464931 | 5.9694580473335e-17 | | postive |
| TNFRSF1B | AL133371.2 | 0.55717698497428 | 2.723186240213e-32 | | postive |
| LEF1 | MEIS1-AS3 | 0.407633667912941 | 1.3219695360104e-16 | | postive |
| DIABLO | MAPKAPK5-AS1 | 0.453215777726157 | 1.35016408661247e-20 | | postive |
| TARDBP | AC005070.3 | 0.41689011895663 | 2.28555400959154e-17 | | postive |
| MLKL | AC107057.1 | -0.483352956188062 | 1.3873423153869e-23 | | negative |
| TNFRSF1B | AC107057.1 | -0.461186052023325 | 2.33706816526045e-21 | | negative |
| CYLD | AC107057.1 | -0.403809839214451 | 2.68692627518482e-16 | | negative |
| CFLAR | LINC01359 | 0.448671684810614 | 3.59583442837294e-20 | | postive |
| TARDBP | AL132989.1 | 0.412491300012813 | 5.29861028217433e-17 | | postive |
| TNFRSF1B | AC012181.2 | 0.424192379313979 | 5.50588551988813e-18 | | postive |
| DIABLO | NRAV | 0.40890261582283 | 1.04260986260923e-16 | | postive |
| USP22 | LINC00239 | -0.433043924017453 | 9.3561925741614e-19 | | negative |
| STAT3 | SNHG19 | -0.410616700099424 | 7.55365355724436e-17 | | negative |
| TARDBP | AL512770.1 | 0.469303032075976 | 3.73371083667343e-22 | | postive |
| BCL2 | AC139100.1 | 0.413935035351269 | 4.02625342788532e-17 | | postive |
| MLKL | AP000695.2 | 0.412010974158256 | 5.80379517951116e-17 | | postive |
| RNF31 | AC245060.2 | 0.419079479506 | 1.49703041190548e-17 | | postive |
| BRAF | NDUFB2-AS1 | 0.406877078633903 | 1.52224377170498e-16 | | postive |
| TNFRSF1B | AL354863.1 | -0.44533226805181 | 7.31804425310558e-20 | | negative |
| STAT3 | RNASEH1-AS1 | -0.400218049369172 | 5.18810645145919e-16 | | negative |
| TARDBP | AC084824.3 | 0.41262685183388 | 5.16403420316512e-17 | | postive |
| TARDBP | LINC00174 | 0.452238604261635 | 1.66880954069603e-20 | | postive |
| FASLG | AC011899.2 | 0.53597066669527 | 1.44057996565394e-29 | | postive |
| MLKL | AC011899.2 | 0.44935300737453 | 3.10756514592614e-20 | | postive |
| TNFRSF1B | AC011899.2 | 0.660194372878692 | 8.49689550114354e-49 | | postive |
| AXL | AC011899.2 | 0.445875891064028 | 6.52214133015186e-20 | | postive |
| FASLG | TRG-AS1 | 0.849632066481767 | 8.37835864367619e-107 | | postive |
| MLKL | TRG-AS1 | 0.571944914525805 | 2.62979913871454e-34 | | postive |
| TNFRSF1B | TRG-AS1 | 0.536995244178587 | 1.07486972578071e-29 | | postive |
| CYLD | TRG-AS1 | 0.458267036092789 | 4.46643816700884e-21 | | postive |
| TARDBP | AC011465.1 | 0.434551579965781 | 6.88218659281071e-19 | | postive |
| MLKL | AL391152.1 | -0.4040883845255 | 2.55241116302138e-16 | | negative |
| CFLAR | AP005131.3 | 0.41746892708223 | 2.044280780047e-17 | | postive |
| AXL | AC009093.2 | 0.409153080474315 | 9.94764135960704e-17 | | postive |
| TSC1 | AL356481.3 | 0.51856812275611 | 1.79388020210464e-27 | | postive |
| TARDBP | AL356481.3 | 0.434855487622779 | 6.46786457900757e-19 | | postive |
| ZBP1 | AL049838.1 | -0.408644750623953 | 1.09422957772233e-16 | | negative |
| TNFSF10 | AL049838.1 | -0.410894626496098 | 7.16781706084562e-17 | | negative |
| BACH2 | AL049838.1 | 0.449763084768313 | 2.84580063486199e-20 | | postive |
| TARDBP | LINC01004 | 0.451526351852551 | 1.94667325361069e-20 | | postive |
| BACH2 | ARHGAP31-AS1 | 0.422660826968837 | 7.44265982483836e-18 | | postive |
| PLK1 | AC026401.3 | 0.496913314019027 | 4.98900903946661e-25 | | postive |
| BNIP3 | AC114803.1 | 0.600752574730367 | 1.5206644703915e-38 | | postive |
| TNFRSF1B | AC138207.1 | 0.488016446331278 | 4.49542303234468e-24 | | postive |
| TSC1 | AC011005.4 | 0.41413912132701 | 3.87253997502383e-17 | | postive |
| SLC39A7 | HCG18 | 0.485193335390324 | 8.91126112165775e-24 | | postive |
| TARDBP | LINC00342 | 0.43871616588428 | 2.92303559018042e-19 | | postive |
| TSC1 | CR936218.1 | 0.428801625713573 | 2.20207091634512e-18 | | postive |
| RNF31 | SNHG9 | -0.52367505418644 | 4.48150852692654e-28 | | negative |
| FADD | AP002761.3 | 0.409499036375465 | 9.32206064538793e-17 | | postive |
| SQSTM1 | AC087289.1 | 0.464679309396368 | 1.06776673784966e-21 | | postive |
| USP22 | AC015922.2 | 0.534321866819609 | 2.30299807850293e-29 | | postive |
| APP | AC015922.2 | 0.401355218965021 | 4.21611772911887e-16 | | postive |
| FASLG | LINC02285 | 0.425935224233694 | 3.89990109914683e-18 | | postive |
| TNFRSF1B | LINC02285 | 0.442545319569712 | 1.31627465629412e-19 | | postive |
| TARDBP | LINC02285 | -0.408824077433991 | 1.05807311267827e-16 | | negative |
| FASLG | AL683807.1 | 0.48531509444117 | 8.65325048730905e-24 | | postive |
| MLKL | AL683807.1 | 0.477376460767576 | 5.73492005003877e-23 | | postive |
| AXL | LNCOG | 0.414580867424157 | 3.5593025264881e-17 | | postive |
| USP22 | PPP4R1-AS1 | -0.402557281408493 | 3.38297574448582e-16 | | negative |
| TNF | AL590369.1 | 0.403185952351879 | 3.01397059330028e-16 | | postive |
| TARDBP | AC007619.1 | 0.479169842069349 | 3.7570648296323e-23 | | postive |
| USP22 | AP001453.2 | -0.480262602424146 | 2.89998750536818e-23 | | negative |
| FASLG | LINC02446 | 0.819819652126008 | 2.60254814054544e-93 | | postive |
| MLKL | LINC02446 | 0.498041389971446 | 3.75792409731981e-25 | | postive |
| TNFRSF1B | LINC02446 | 0.42592682191749 | 3.90640926347286e-18 | | postive |
| TARDBP | AC020913.3 | 0.445573200689667 | 6.95415006806106e-20 | | postive |
| CFLAR | AC124319.2 | 0.475613605574177 | 8.67017848029372e-23 | | postive |
| APP | GNG12-AS1 | 0.42740172019188 | 2.91309366970721e-18 | | postive |
| IDH2 | ZNF710-AS1 | 0.547095723355508 | 5.67512581165026e-31 | | postive |
| MLKL | AC007991.4 | 0.403053095274082 | 3.08850389552124e-16 | | postive |
| ZBP1 | AC007991.4 | 0.407405072005558 | 1.37958286843421e-16 | | postive |
| TNFRSF1A | AC006064.2 | 0.407208576641394 | 1.43107111705312e-16 | | postive |
| FASLG | AC145098.1 | 0.485994040719254 | 7.34403201729278e-24 | | postive |
| MLKL | AC145098.1 | 0.501576896933396 | 1.5355764655639e-25 | | postive |
| TNFRSF1B | AC145098.1 | 0.636399359398442 | 2.01354765030143e-44 | | postive |
| CYLD | AC145098.1 | 0.403856497313967 | 2.66391866348707e-16 | | postive |
| STAT3 | AC145098.1 | 0.438113855790891 | 3.31083178082063e-19 | | postive |
| AXL | AC145098.1 | 0.439563906265321 | 2.45189757009598e-19 | | postive |
| DIABLO | NEAT1 | -0.430191835009362 | 1.66567280153955e-18 | | negative |
| CFLAR | NEAT1 | 0.472062984187245 | 1.97890966410101e-22 | | postive |
| FASLG | LINC01857 | 0.513121815849277 | 7.6748462589598e-27 | | postive |
| MLKL | LINC01857 | 0.442062304960663 | 1.45644775701259e-19 | | postive |
| TARDBP | AC120053.1 | 0.457351400885529 | 5.46564112243751e-21 | | postive |
| FASLG | AC079015.1 | 0.484934092845872 | 9.48614636797185e-24 | | postive |
| MAP3K7 | LINC01611 | 0.425062277638956 | 4.63638895536112e-18 | | postive |
| USP22 | AL162171.1 | 0.418207001597207 | 1.7726449702011e-17 | | postive |
| APP | AL162171.1 | 0.487134380423995 | 5.57077490067753e-24 | | postive |
| MAP3K7 | MANEA-DT | 0.43943711040961 | 2.51728748880008e-19 | | postive |
| TARDBP | AL109811.1 | 0.525222491486791 | 2.93007913735748e-28 | | postive |
| STAT3 | ZFAS1 | -0.409277383424622 | 9.71826194827003e-17 | | negative |
| TNFRSF1B | AL512625.1 | -0.402899810033114 | 3.17674727546159e-16 | | negative |
| PLK1 | AC012073.1 | 0.442521213315949 | 1.32294428557674e-19 | | postive |
| FASLG | PSMB8-AS1 | 0.519810820794997 | 1.28278102050082e-27 | | postive |
| MLKL | PSMB8-AS1 | 0.604901431622027 | 3.43422099818357e-39 | | postive |
| TLR3 | PSMB8-AS1 | 0.561952108473631 | 6.23043240361164e-33 | | postive |
| ZBP1 | PSMB8-AS1 | 0.611074910333779 | 3.60192202203777e-40 | | postive |
| TNFSF10 | PSMB8-AS1 | 0.552904541539633 | 9.98854833616574e-32 | | postive |
| TNFRSF1B | PSMB8-AS1 | 0.414967271285443 | 3.30581587099127e-17 | | postive |
| CYLD | PSMB8-AS1 | 0.451357189070783 | 2.01909043550497e-20 | | postive |
| USP22 | PSMB8-AS1 | -0.403152212985321 | 3.03272950902655e-16 | | negative |
| DDX58 | PSMB8-AS1 | 0.475718192358363 | 8.46072521955395e-23 | | postive |
| APP | PSMB8-AS1 | -0.416580339122699 | 2.42596278243704e-17 | | negative |
| BACH2 | AC008555.1 | 0.440527593600429 | 2.00664395886023e-19 | | postive |
| USP22 | AC015912.3 | -0.431732535511127 | 1.22058698357205e-18 | | negative |
| AXL | AC015912.3 | -0.400643708448072 | 4.80092639788895e-16 | | negative |
| TSC1 | AL031600.1 | 0.40169424560343 | 3.9626457989419e-16 | | postive |
| CFLAR | AP006621.2 | 0.410958203665033 | 7.08231332739238e-17 | | postive |
| TARDBP | AC127024.4 | 0.43106425169141 | 1.39707347117298e-18 | | postive |
| USP22 | LINC02004 | -0.445846867058127 | 6.56239288468213e-20 | | negative |
| CFLAR | AL157871.2 | 0.425141039160069 | 4.56468220997584e-18 | | postive |
| ALK | AC106870.1 | 0.692172536653736 | 2.39763480741198e-55 | | postive |
| RNF31 | LINC02482 | -0.408826586880402 | 1.05757556787202e-16 | | negative |
| MLKL | AL731567.1 | 0.406070609193386 | 1.76853906085494e-16 | | postive |
| TNFRSF1B | AL731567.1 | 0.440095042360339 | 2.19567947358482e-19 | | postive |
| TNFRSF1B | AC012181.1 | 0.421379212758052 | 9.56654954532557e-18 | | postive |
| BRAF | AC004918.3 | 0.420543975957229 | 1.12605336511069e-17 | | postive |
| SIRT2 | AC104534.1 | 0.441540994340039 | 1.62423610956405e-19 | | postive |
| TARDBP | LINC02604 | 0.476872067782401 | 6.45643515710365e-23 | | postive |
| TARDBP | AC008735.2 | 0.421097865506743 | 1.01071183894221e-17 | | postive |
| MLKL | AC007991.2 | 0.402094105856694 | 3.68287043856965e-16 | | postive |
| ZBP1 | AC007991.2 | 0.446839238856837 | 5.31565372489506e-20 | | postive |
| TNFSF10 | AC007991.2 | 0.422140633899128 | 8.24208712785135e-18 | | postive |
| CYLD | AL121583.1 | -0.400461083450834 | 4.96343914982615e-16 | | negative |
| CYLD | PXN-AS1 | -0.404748837369372 | 2.25933077391235e-16 | | negative |
| DIABLO | U62317.2 | -0.404936254978313 | 2.18236186714457e-16 | | negative |
| ATRX | AC060766.6 | 0.421335259717495 | 9.6490858554782e-18 | | postive |
| TNFRSF1B | VPS33B-DT | -0.465802159183948 | 8.28502906853729e-22 | | negative |
| AXL | FAM198B-AS1 | 0.428378983924127 | 2.39651184437226e-18 | | postive |
| TLR3 | AC108134.2 | 0.443708931014379 | 1.03082249921668e-19 | | postive |
| TARDBP | AC010976.1 | 0.482225089222492 | 1.81729935446147e-23 | | postive |
| SIRT2 | AC011445.1 | 0.427181502389357 | 3.04382558822072e-18 | | postive |
| SPATA2 | AC011445.1 | 0.407439673919018 | 1.3707063530849e-16 | | postive |
| CDKN2A | AL449423.1 | 0.565509797698251 | 2.04400129476723e-33 | | postive |
| TARDBP | AL513320.1 | 0.40525110596182 | 2.05881401371042e-16 | | postive |
| MPG | AC010468.1 | 0.50425949312008 | 7.73259166816113e-26 | | postive |
| TARDBP | AC010468.1 | -0.465763702471122 | 8.35746224528966e-22 | | negative |
| TSC1 | AP002490.1 | 0.424071540706168 | 5.63870597117829e-18 | | postive |
| FASLG | HCP5 | 0.506146140645314 | 4.7555259240642e-26 | | postive |
| MLKL | HCP5 | 0.592378375497348 | 2.87074728266196e-37 | | postive |
| TLR3 | HCP5 | 0.488081343653207 | 4.42493424165091e-24 | | postive |
| ZBP1 | HCP5 | 0.563878126922185 | 3.41352184586925e-33 | | postive |
| TNFSF10 | HCP5 | 0.552109644392066 | 1.26946334752478e-31 | | postive |
| TNFRSF1B | HCP5 | 0.412534514344532 | 5.25533716617626e-17 | | postive |
| CYLD | HCP5 | 0.481644674271264 | 2.08733455712505e-23 | | postive |
| DDX58 | HCP5 | 0.500444449229256 | 2.04767091345211e-25 | | postive |
| TARDBP | AC124944.3 | 0.442201209894547 | 1.41469705933205e-19 | | postive |
| PLK1 | AL023803.2 | 0.46286501506707 | 1.60565118554719e-21 | | postive |
| FAS | AL157394.1 | 0.747509208862188 | 5.73218470773407e-69 | | postive |
| TNFSF10 | AL513534.2 | -0.451756707204627 | 1.85215782439754e-20 | | negative |
| TNFRSF1B | AL513534.2 | -0.41725017890751 | 2.13236994110592e-17 | | negative |
| SQSTM1 | AL513534.2 | -0.450207355323971 | 2.58668685047229e-20 | | negative |
| TARDBP | AC109460.2 | 0.432557009698515 | 1.0328356643465e-18 | | postive |
| RNF31 | AC026979.2 | -0.4492500127183 | 3.17694989794951e-20 | | negative |
| TSC1 | AC012615.6 | 0.450799182701489 | 2.27729049841251e-20 | | postive |
| FADD | AC007849.1 | -0.400806699164771 | 4.66030384485105e-16 | | negative |
| TARDBP | AC007849.1 | 0.510229255171324 | 1.64343242816477e-26 | | postive |
| FASLG | AC099524.1 | 0.464563648946725 | 1.09598034354086e-21 | | postive |
| MLKL | AC099524.1 | 0.428884812750876 | 2.16566958312883e-18 | | postive |
| SIRT2 | AC008982.2 | 0.439496990837733 | 2.48619544078473e-19 | | postive |
| MYC | VPS9D1-AS1 | 0.506593327783103 | 4.23606834226466e-26 | | postive |
| TARDBP | PRC1-AS1 | 0.408179959330739 | 1.19368606965458e-16 | | postive |
| TARDBP | IGF2BP2-AS1 | 0.435300523048839 | 5.90505876961339e-19 | | postive |
| TARDBP | AL008729.1 | 0.43048257076794 | 1.57095424997792e-18 | | postive |
| MLKL | GIHCG | -0.414115241952365 | 3.89022322959569e-17 | | negative |
| TNFRSF1B | GIHCG | -0.487150628186715 | 5.54884133848067e-24 | | negative |
| CYLD | GIHCG | -0.403125450373598 | 3.04769085788267e-16 | | negative |
| TARDBP | PSMA3-AS1 | 0.422654498074442 | 7.45191193212465e-18 | | postive |
| FASLG | LINC00582 | 0.41999888841483 | 1.2521613309515e-17 | | postive |
| FAS | LINC01614 | 0.417877778102822 | 1.88912166925933e-17 | | postive |
| AXL | LINC01614 | 0.500199562239862 | 2.17884930616458e-25 | | postive |
| TSC1 | AC108010.1 | 0.459741045328008 | 3.22297182487555e-21 | | postive |
| RNF31 | AC108010.1 | 0.479765470113593 | 3.26290564052217e-23 | | postive |
| TSC1 | AC005519.1 | 0.450505160520208 | 2.42615089597326e-20 | | postive |
| TARDBP | AC005519.1 | 0.437353647447832 | 3.87319879842494e-19 | | postive |
| FASLG | LINC02084 | 0.7378765773038 | 2.37936683877302e-66 | | postive |
| MLKL | LINC02084 | 0.441545770316823 | 1.62261578443478e-19 | | postive |
| RNF31 | AC027644.3 | -0.419151662970292 | 1.4762133165805e-17 | | negative |
| FASLG | MIAT | 0.424249128392494 | 5.4445756189151e-18 | | postive |
| MLKL | MIAT | 0.446073342081157 | 6.25468698974201e-20 | | postive |
| CFLAR | HEXD-IT1 | 0.413809269810365 | 4.1239461751957e-17 | | postive |
| MLKL | AC023825.2 | 0.482059727544874 | 1.89051221746823e-23 | | postive |
| TSC1 | AC138956.2 | 0.404978342985236 | 2.16543436506409e-16 | | postive |
| USP22 | PRR34-AS1 | -0.406733793643244 | 1.56339527203819e-16 | | negative |
| APP | PRR34-AS1 | -0.405730461884248 | 1.88378814592373e-16 | | negative |
| CFLAR | RUSC1-AS1 | 0.474971700018581 | 1.00723682791711e-22 | | postive |
| TARDBP | RUSC1-AS1 | 0.407865269147306 | 1.26600969221748e-16 | | postive |
| SQSTM1 | LINC02159 | 0.443652552258266 | 1.04312664428871e-19 | | postive |
| USP22 | AL731577.2 | 0.474760795762232 | 1.05801555880431e-22 | | postive |
| RNF31 | AL731577.2 | 0.516280155293365 | 3.3141741249589e-27 | | postive |
| STAT3 | AC003070.1 | 0.413344637341766 | 4.50547961601479e-17 | | postive |
| RNF31 | AC009034.1 | -0.411194903543331 | 6.77269875892127e-17 | | negative |
| MPG | AC020663.2 | 0.414270038184024 | 3.7769871240759e-17 | | postive |
| TSC1 | AC114730.3 | 0.433985915744505 | 7.7240529183536e-19 | | postive |
| DNMT1 | AC091057.1 | 0.435075602291291 | 6.18320061919566e-19 | | postive |
| PLK1 | AC091057.1 | 0.410109007714131 | 8.31183514362292e-17 | | postive |
| MPG | AC091057.1 | -0.436667900543881 | 4.46048548391596e-19 | | negative |
| TARDBP | AC091057.1 | 0.441973253097216 | 1.4838496000338e-19 | | postive |
| TARDBP | AC002553.1 | 0.504469726884555 | 7.32594549918795e-26 | | postive |
| MLKL | ZNF197-AS1 | -0.407937791524486 | 1.2489699078633e-16 | | negative |
| USP22 | AC145343.1 | -0.469036547220047 | 3.96853273026963e-22 | | negative |
| CFLAR | MALAT1 | 0.459813107790805 | 3.17183879040524e-21 | | postive |
| ATRX | MALAT1 | 0.436782970678683 | 4.35615645035883e-19 | | postive |
| TARDBP | MALAT1 | 0.500454551868598 | 2.04243003473734e-25 | | postive |
| MLKL | AC093278.2 | 0.439244445442463 | 2.61994493721503e-19 | | postive |
| TNFRSF1B | AC093278.2 | 0.415791162185657 | 2.82309689562756e-17 | | postive |
| AXL | AC093278.2 | 0.40952206030712 | 9.28182680650366e-17 | | postive |
| USP22 | EXOSC10-AS1 | -0.400298683080082 | 5.11248181397695e-16 | | negative |
| BNIP3 | AL162274.1 | 0.44060949337628 | 1.97270001026183e-19 | | postive |
| TARDBP | ZDHHC20-IT1 | 0.40092840985359 | 4.55794058582482e-16 | | postive |
| TARDBP | AC005776.2 | 0.441116256832281 | 1.77488722812908e-19 | | postive |
| RIPK3 | TNFRSF14-AS1 | 0.400811470928223 | 4.65624844700618e-16 | | postive |
| FASLG | AC243960.1 | 0.732390876664348 | 6.55278683076094e-65 | | postive |
| MLKL | AC243960.1 | 0.532068639782099 | 4.35450991896976e-29 | | postive |
| TNFRSF1B | AC243960.1 | 0.494679819187288 | 8.71628927848041e-25 | | postive |
| TSC1 | AC037459.3 | 0.45134093076639 | 2.02618864621172e-20 | | postive |
| ATRX | Z83843.1 | 0.426687221644619 | 3.35862311012581e-18 | | postive |
| RNF31 | AC074135.1 | 0.419129162805746 | 1.48267146519161e-17 | | postive |
| FASLG | AL023653.1 | 0.415511464332691 | 2.97865058669675e-17 | | postive |
| USP22 | CYTOR | -0.453229137185163 | 1.3462522471723e-20 | | negative |
| MPG | CYTOR | 0.41345684292265 | 4.4102822957229e-17 | | postive |
| RNF31 | CYTOR | -0.419472484166245 | 1.38708157054418e-17 | | negative |
| TLR3 | AC107308.1 | -0.434507549807005 | 6.94433973951267e-19 | | negative |
| SQSTM1 | AC107308.1 | -0.411923521905812 | 5.90073438933498e-17 | | negative |
| MLKL | C10orf55 | 0.401422666196976 | 4.16445457080574e-16 | | postive |
| TARDBP | AC016949.1 | 0.478730367949909 | 4.16830902529789e-23 | | postive |
| SIRT2 | AC011479.3 | 0.449373081802678 | 3.09421634419662e-20 | | postive |
| AXL | LINC00702 | 0.497277459274391 | 4.55342476622179e-25 | | postive |
| KLF9 | LINC00702 | 0.40763974405635 | 1.32047084788057e-16 | | postive |
| RNF31 | ARF4-AS1 | -0.41529910189217 | 3.10235133475256e-17 | | negative |
| FAS | LINC01094 | 0.454465486317583 | 1.02865422270304e-20 | | postive |
| FASLG | LINC01094 | 0.548048844966007 | 4.27748721801271e-31 | | postive |
| MLKL | LINC01094 | 0.576310721147304 | 6.37505462271241e-35 | | postive |
| TLR3 | LINC01094 | 0.407158024404349 | 1.44461977271285e-16 | | postive |
| TNFSF10 | LINC01094 | 0.43870494061483 | 2.9298366562406e-19 | | postive |
| TNFRSF1B | LINC01094 | 0.596173875827577 | 7.66157947912629e-38 | | postive |
| CYLD | LINC01094 | 0.4814904494298 | 2.16550738307176e-23 | | postive |
| AXL | LINC01094 | 0.431554967988623 | 1.2652176791801e-18 | | postive |
| TARDBP | LINC01094 | -0.407154381126623 | 1.44560107000866e-16 | | negative |
| FASLG | LINC01679 | 0.457019060813669 | 5.88030956565903e-21 | | postive |
| MLKL | LINC01679 | 0.507586607529128 | 3.27427868624407e-26 | | postive |
| TNFRSF1B | LINC01679 | 0.468285247703008 | 4.71179441583039e-22 | | postive |
| STUB1 | LINC00235 | 0.476568100600265 | 6.93372709397221e-23 | | postive |
| RNF31 | AC016876.2 | -0.457842078420061 | 4.90554714426683e-21 | | negative |
| FASLG | HLA-DQB1-AS1 | 0.499589143212769 | 2.54303392022843e-25 | | postive |
| MLKL | HLA-DQB1-AS1 | 0.455407677043057 | 8.37328117089895e-21 | | postive |
| RIPK3 | HLA-DQB1-AS1 | 0.400811403020556 | 4.65630613566568e-16 | | postive |
| TLR3 | HLA-DQB1-AS1 | 0.415368189324411 | 3.06156405049591e-17 | | postive |
| TNFRSF1B | HLA-DQB1-AS1 | 0.430691128774844 | 1.50629171138276e-18 | | postive |
| MLKL | AC017002.3 | 0.42472393345612 | 4.95723337364342e-18 | | postive |
| BNIP3 | ITGA6-AS1 | 0.402844766589691 | 3.20903486663942e-16 | | postive |
| TARDBP | PRKCZ-AS1 | 0.546431413393214 | 6.90754468840141e-31 | | postive |
| CFLAR | SSBP3-AS1 | 0.412722103878256 | 5.07148237590882e-17 | | postive |
| MPG | SSBP3-AS1 | -0.418138749663452 | 1.79619704516309e-17 | | negative |
| TARDBP | SSBP3-AS1 | 0.494565825919482 | 8.96708637860211e-25 | | postive |
| TNFRSF1B | ITGB2-AS1 | 0.4268430032044 | 3.25610261330384e-18 | | postive |
| TARDBP | AC024560.3 | 0.495890497521524 | 6.444707282483e-25 | | postive |
| CFLAR | AL135999.1 | 0.447997441277582 | 4.15317870850803e-20 | | postive |
| RNF31 | AL135999.1 | 0.408588189342145 | 1.10588334992008e-16 | | postive |
| TNFRSF1B | AC002310.1 | -0.404211379591318 | 2.49513980003998e-16 | | negative |
| TARDBP | AL365330.1 | 0.541734193967116 | 2.73785387061645e-30 | | postive |
| TARDBP | AC011462.5 | 0.419134955910398 | 1.48100603992856e-17 | | postive |
| TSC1 | AC093110.1 | 0.431436726870604 | 1.29582330001653e-18 | | postive |
| FASLG | LINC00996 | 0.625883895229047 | 1.31011513339579e-42 | | postive |
| MLKL | LINC00996 | 0.499593118837418 | 2.54047782417731e-25 | | postive |
| TNFRSF1B | LINC00996 | 0.406354827442643 | 1.67757264624581e-16 | | postive |
| TARDBP | AC016405.1 | 0.419674426649462 | 1.33371248034292e-17 | | postive |
| MLKL | AC083862.2 | 0.503963776489662 | 8.3425452985513e-26 | | postive |
| TLR3 | AC083862.2 | 0.473895903827097 | 1.29399917784672e-22 | | postive |
| ZBP1 | AC083862.2 | 0.61728618804354 | 3.54204390077667e-41 | | postive |
| TNFSF10 | AC083862.2 | 0.412013552734452 | 5.80096074857962e-17 | | postive |
| CYLD | AC083862.2 | 0.411699942573039 | 6.15585741096677e-17 | | postive |
| DDX58 | AC083862.2 | 0.483849098456389 | 1.2316049492075e-23 | | postive |
| CFLAR | FMR1-IT1 | 0.448056023918766 | 4.10155651010741e-20 | | postive |
| DIABLO | AC107375.1 | -0.423989412710411 | 5.73077026201682e-18 | | negative |
| TARDBP | AC107375.1 | 0.420625400099757 | 1.1083202109906e-17 | | postive |
| FASLG | AC022126.1 | 0.701215846415237 | 2.34155151381656e-57 | | postive |
| FASLG | AC090559.1 | 0.507101911272081 | 3.71313019270513e-26 | | postive |
| MLKL | AC090559.1 | 0.449370148806225 | 3.09616315781993e-20 | | postive |
| TNFRSF1B | AC090559.1 | 0.664603221166776 | 1.18687600578261e-49 | | postive |
| CYLD | AC090559.1 | 0.447054728014158 | 5.07746622008574e-20 | | postive |
| AXL | AC090559.1 | 0.553006842232059 | 9.68461768836538e-32 | | postive |
| TNFRSF1B | AL133215.2 | -0.405185594711967 | 2.08394147974998e-16 | | negative |
| STAT3 | AL133215.2 | -0.402067383587674 | 3.70094695953735e-16 | | negative |
| FASLG | LINC00892 | 0.616801342515839 | 4.25294863490849e-41 | | postive |
| MLKL | LINC00892 | 0.425359123984968 | 4.37177258589743e-18 | | postive |
| MYC | PVT1 | 0.567631679832853 | 1.04469662768629e-33 | | postive |
| CFLAR | AC087284.1 | 0.432630159902523 | 1.01762054799592e-18 | | postive |
| USP22 | KMT2E-AS1 | -0.404149070752311 | 2.52399378030806e-16 | | negative |
| FASLG | AL365361.1 | 0.597220723369767 | 5.30573728951456e-38 | | postive |
| MLKL | AL365361.1 | 0.463983046859837 | 1.24909321510796e-21 | | postive |
| TNFRSF1B | AL365361.1 | 0.417842117151307 | 1.90218095203248e-17 | | postive |
| MYC | SNHG4 | 0.497690527107067 | 4.10462979514818e-25 | | postive |
| HSPA4 | SNHG4 | 0.451953386092923 | 1.7750472484711e-20 | | postive |
| MYC | AC091563.1 | 0.430729014413457 | 1.49482969125865e-18 | | postive |
| TARDBP | AC015849.3 | 0.468177629643315 | 4.82893005233196e-22 | | postive |
| CFLAR | AL159169.2 | 0.411760841036883 | 6.08531270671388e-17 | | postive |
| ATRX | AC069549.1 | 0.401951494210515 | 3.78035446301115e-16 | | postive |
| FASLG | AC015819.1 | 0.515017441109838 | 4.64128601065675e-27 | | postive |
| MLKL | AC015819.1 | 0.48988228100686 | 2.8500256855623e-24 | | postive |
| TNFRSF1B | AC015819.1 | 0.480221762348458 | 2.92823627913258e-23 | | postive |
| TARDBP | Z94721.1 | 0.409081214297207 | 1.00826749052768e-16 | | postive |
| FASLG | CARD8-AS1 | 0.430382402746968 | 1.60297390698021e-18 | | postive |
| USP22 | AC004888.1 | -0.400100288401509 | 5.30052830744187e-16 | | negative |
| BACH2 | AC004888.1 | -0.454151232882009 | 1.10158260589175e-20 | | negative |
| FADD | AP000487.1 | 0.42375814278862 | 5.9980341784748e-18 | | postive |
| SQSTM1 | AC010615.2 | -0.401016939418353 | 4.4848735516088e-16 | | negative |
| FASLG | LINC01943 | 0.536057620956588 | 1.40527252297597e-29 | | postive |
| MLKL | LINC01943 | 0.520034436992603 | 1.20748463418072e-27 | | postive |
| FASLG | AC004585.1 | 0.670465565109459 | 8.22030830807795e-51 | | postive |
| MLKL | AC004585.1 | 0.453641798972182 | 1.23076582799146e-20 | | postive |
| TNFRSF1B | AC004585.1 | 0.400860191667736 | 4.61503967885376e-16 | | postive |
| ATRX | LINC01278 | 0.430913282624703 | 1.44029262826913e-18 | | postive |
| TSC1 | AL445931.1 | 0.495811505984743 | 6.57316746998142e-25 | | postive |
| FASLG | AC026369.3 | 0.492104636374803 | 1.65013246490025e-24 | | postive |
| TSC1 | AL354733.3 | 0.43810084435763 | 3.31974472552541e-19 | | postive |
| SIRT2 | ERVK9-11 | 0.480787551193468 | 2.55995056507976e-23 | | postive |
| HSP90AA1 | AL049840.3 | 0.424006394529314 | 5.71161349286817e-18 | | postive |
| MAP3K7 | AL513550.1 | 0.461912229662821 | 1.98734401935813e-21 | | postive |
| ZBP1 | AL445490.1 | 0.628791074667218 | 4.1969564734282e-43 | | postive |
| DDX58 | AL445490.1 | 0.690878450456688 | 4.58552207834792e-55 | | postive |
