# Supplementary material for: Necroptosis-associated long noncoding RNAs can predict prognosis and differentiate between cold and hot tumors in ovarian cancer
Source: Front Oncol. 2022 Jul 28;12:967207. doi: 10.3389/fonc.2022.967207 (PMC9366220; doi:10.3389/fonc.2022.967207)
Supplement: Appendix D2 — (Table 2): The data of immune infiltrating cell from different platforms in risk model [file Table_2.docx]

| immune | cor | pvalue |
| --- | --- | --- |
| B cell_TIMER | -0.214364595226505 | 0.000540009408879218 |
| Macrophage_TIMER | 0.270713199644809 | 1.07671623440101e-05 |
| B cell plasma_CIBERSORT | -0.16689529744479 | 0.0073331434293921 |
| T cell follicular helper_CIBERSORT | -0.253783246119195 | 3.85151180561204e-05 |
| NK cell activated_CIBERSORT | -0.133703008373586 | 0.0321450083376053 |
| Macrophage M1_CIBERSORT | -0.275054017585736 | 7.65680303105727e-06 |
| Macrophage M2_CIBERSORT | 0.212052546903084 | 0.000621882453285634 |
| Myeloid dendritic cell activated_CIBERSORT | -0.196748550171214 | 0.00152552759228507 |
| B cell plasma_CIBERSORT-ABS | -0.183991167920408 | 0.00307100116696247 |
| T cell follicular helper_CIBERSORT-ABS | -0.253210616135723 | 4.01510900930669e-05 |
| Macrophage M1_CIBERSORT-ABS | -0.21225603181958 | 0.000614240540965346 |
| Myeloid dendritic cell activated_CIBERSORT-ABS | -0.184031516148363 | 0.00306442205941399 |
| Monocyte_QUANTISEQ | -0.162503653541967 | 0.00905945753319393 |
| Neutrophil_QUANTISEQ | 0.132786662756953 | 0.0333548014987162 |
| T cell CD8+_QUANTISEQ | -0.124038692872378 | 0.0469804946689253 |
| cytotoxicity score_MCPCOUNTER | -0.205926079883998 | 0.000897546900772793 |
| NK cell_MCPCOUNTER | -0.128645830019537 | 0.0393157143168477 |
| Monocyte_MCPCOUNTER | 0.140110439744204 | 0.0246858580004437 |
| Macrophage/Monocyte_MCPCOUNTER | 0.140110439744204 | 0.0246858580004437 |
| Neutrophil_MCPCOUNTER | 0.209973369189895 | 0.000705165788461659 |
| Endothelial cell_MCPCOUNTER | 0.200266237406618 | 0.00124824046053655 |
| Cancer associated fibroblast_MCPCOUNTER | 0.146314633796351 | 0.0189348360078458 |
| Myeloid dendritic cell activated_XCELL | -0.130150311835908 | 0.0370534717779546 |
| T cell CD8+ central memory_XCELL | -0.208230509225657 | 0.000782791017540323 |
| T cell CD8+ effector memory_XCELL | -0.158943907528254 | 0.0107141442533442 |
| Class-switched memory B cell_XCELL | -0.124275330839511 | 0.0465584063811879 |
| Cancer associated fibroblast_XCELL | 0.219120264665457 | 0.00040203704324668 |
| Granulocyte-monocyte progenitor_XCELL | 0.160259062359805 | 0.0100740375174077 |
| Hematopoietic stem cell_XCELL | 0.140661755947042 | 0.0241200852920514 |
| Mast cell_XCELL | 0.138672601281277 | 0.0262154521553791 |
| Plasmacytoid dendritic cell_XCELL | -0.320957090081044 | 1.43998477475254e-07 |
| B cell plasma_XCELL | -0.128848240481594 | 0.0390047283951439 |
| T cell CD4+ Th1_XCELL | -0.286740840303998 | 2.96988542394404e-06 |
| T cell CD4+ Th2_XCELL | -0.166068350811509 | 0.00763382207651536 |
| stroma score_XCELL | 0.216533188361299 | 0.000472401830880099 |
| Cancer associated fibroblast_EPIC | 0.13696451181211 | 0.0281377272200502 |
| Endothelial cell_EPIC | 0.191371391284908 | 0.00205966275907327 |
| NK cell_EPIC | -0.176773578729895 | 0.00447610930511409 |
| uncharacterized cell_EPIC | -0.167664526728765 | 0.00706298081299182 |
