# Supplementary material for: Necroptosis-associated long noncoding RNAs can predict prognosis and differentiate between cold and hot tumors in ovarian cancer
Source: Front Oncol. 2022 Jul 28;12:967207. doi: 10.3389/fonc.2022.967207 (PMC9366220; doi:10.3389/fonc.2022.967207)
Supplement: Appendix D3 — (Table 3): The data of immune infiltrating cell from different platforms in cluster [file Table_3.docx]

| immune | pvalue |
| --- | --- |
| B cell_TIMER | 0.00032018480509673 |
| Myeloid dendritic cell_TIMER | 0.0239861038397503 |
| T cell CD8+_CIBERSORT | 0.0171562014492928 |
| T cell regulatory (Tregs)_CIBERSORT | 0.0446626733842584 |
| Macrophage M1_CIBERSORT | 7.990340127044e-05 |
| T cell CD8+_CIBERSORT-ABS | 0.0260832597306329 |
| NK cell activated_CIBERSORT-ABS | 0.0117385510102505 |
| Macrophage M1_CIBERSORT-ABS | 0.00110356246877934 |
| Macrophage M1_QUANTISEQ | 0.0257679053791559 |
| Monocyte_QUANTISEQ | 0.0155593826776356 |
| Neutrophil_QUANTISEQ | 0.0337710459668052 |
| Myeloid dendritic cell_QUANTISEQ | 0.0121231521709385 |
| uncharacterized cell_QUANTISEQ | 0.0216222316212705 |
| cytotoxicity score_MCPCOUNTER | 1.6741861157224e-06 |
| NK cell_MCPCOUNTER | 0.00205749015518637 |
| Neutrophil_MCPCOUNTER | 0.000183271426261938 |
| Endothelial cell_MCPCOUNTER | 0.000346881681561467 |
| Myeloid dendritic cell activated_XCELL | 1.16115789860572e-05 |
| B cell_XCELL | 0.0335202043300708 |
| T cell CD4+ naive_XCELL | 0.0140301500454899 |
| T cell CD4+ effector memory_XCELL | 0.0292006813765503 |
| T cell CD8+_XCELL | 0.0310752289809937 |
| T cell CD8+ central memory_XCELL | 4.5516506990522e-05 |
| Class-switched memory B cell_XCELL | 0.00186362247465001 |
| Common lymphoid progenitor_XCELL | 0.0409174817795329 |
| Myeloid dendritic cell_XCELL | 0.00290251370469788 |
| Macrophage_XCELL | 0.00701233652664861 |
| Macrophage M1_XCELL | 0.00977979907975943 |
| Monocyte_XCELL | 0.0183390065810117 |
| Plasmacytoid dendritic cell_XCELL | 2.56787976664733e-09 |
| immune score_XCELL | 0.00492991893406277 |
| T cell CD8+_EPIC | 0.0139428668151084 |
| Endothelial cell_EPIC | 0.000983396102143988 |
| NK cell_EPIC | 9.44499800388303e-06 |
